# A meta‐analysis: Does vitamin D play a promising role in sleep disorders?

**DOI:** 10.1002/fsn3.1867

**Published:** 2020-09-09

**Authors:** Shoumeng Yan, Zhenwei Tian, Hantong Zhao, Changcong Wang, Yingan Pan, Nan Yao, Yinpei Guo, Han Wang, Bo Li, Weiwei Cui

**Affiliations:** ^1^ Department of Epidemiology and Biostatistics School of Public Health Jilin University Changchun P. R. China; ^2^ Department of Emergency and critical care The Second Hospital of Jilin University Changchun P. R. China; ^3^ Department of Nutrition and Food Hygiene School of Public Health Jilin University Changchun P. R. China

**Keywords:** meta‐analysis, PSQI, sleep disorders, sleep quality, vitamin D

## Abstract

**Background:**

Sleep disorders, one of the most common problems in the general population, have been related to a series of harmful health consequences. Vitamin D appears to be associated with sleep disorders. However, the difference in vitamin D levels between sleep disorder subjects and people without a sleep disorder is unclear. Simultaneously, the influence of vitamin D replenishment on sleep disorders remains controversial.

**Methods:**

PubMed, MEDLINE, Web of Science, and Cochrane Library were searched for literatures published until October 2019. Using a random effects model, a meta‐analysis was conducted to calculate the standard mean difference to evaluate the difference in vitamin D concentrations between sleep disorder subjects and normal people and the efficacy of vitamin D supplementation on sleep disorders.

**Results:**

Our study found that the serum vitamin D levels in the sleep disorder subjects were lower than that in the normal people (SMD = −0.75 ng/ml, 95% CI = −0.93, −0.57 ng/ml). Moreover, the Pittsburgh Sleep Quality Index (PSQI)in the subjects with vitamin D supplementation was lower than that in the controls (SMD = −0.45, 95% CI = −0.76, −0.13).

**Conclusions:**

Vitamin D could play a promising role in sleep disorders. More data are required to confirm the efficacy of vitamin D supplementation for improving sleep disorders.

## Graphical abstract

The serum vitamin D levels in the sleep disorder subjects were lower than that in the normal people. Meanwhile, the Pittsburgh Sleep Quality Index (PSQI)in the subjects with vitamin D supplementation was lower than that in the controls.

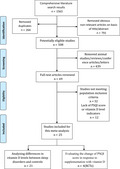

**Abbreviations**
CI: chemiluminescence immunoassay
CIs: confidence intervals
EI: electrochemiluminescence immunoassay
NOS: Newcastle–Ottawa Scale
OSAS: obstructive sleep apnea syndrome
PSQI: Pittsburgh Sleep Quality Index
RCTs: randomized control trials
SMD: standard mean difference

## 1 INTRODUCTION

Sleep is a complex physiological state that involves a period of intense metabolic activity (de Oliveira, Hirotsu, Tufik, & Andersen, 2017). Shortening or interrupting sleep may cause some nonspecific symptoms, including general weakness, physical discomfort, cognitive, and emotional impairment (McCarty, Chesson, Jain, & Marino, 2014). Most sleep disorders, such as sleep apnea, periodic leg dyskinesia, and restless legs syndrome, cause sleep deprivation (Tufik, Andersen, Bittencourt, & Mello, 2009). Sleep disorders have been related to a series of adverse health consequences, involving an elevated risk of hypertension, diabetes, and other chronic diseases (Institute of Medicine Committee on Sleep & Research, 2006; Riemann, 2009). Studies have shown that the prevalence of sleep disorders tends to rise with age. Almost 41% of elderly people have sleep disorders with insomnia (Tsou, 2013). Young people today also experience a number of sleep disorders, which may impact academic performance, health, and mood (Gaultney, 2010). Similarly, sleep problems are currently common in children, with approximately 25% have experienced sleep problems (McDonagh, Holmes, & Hsu, 2019).

As a fat‐soluble vitamin, vitamin D not only plays a role in regulating bone homeostasis but also is involved in the presentation and severity of sleep disorders (Archontogeorgis, Nena, Papanas, & Steiropoulos, 2018; Kulie, Groff, Redmer, Hounshell, & Schrager, 2010). Vitamin D target neurons are supposed to be participated in sleep regulation, and its receptors have been found in the hypothalamus and other brain regions, which are related to the regulation of sleep–wake cycle (Gominak & Stumpf, 2012; Saper, Scammell, & Lu, 2005). Previous studies have shown that abnormally low concentrations of vitamin D are general in patients seeking sleep medication and may be the causes or contributors to sleep disorder (McCarty et al., 2014). Furthermore, Majid's study has shown that the vitamin D supplementation improves sleep quality and raises sleep duration in subjects with sleep disorders (Majid, Ahmad, Bizhan, Hosein, & Mohammad, 2018). However, the results of other studies are not consistent with it (Gunduz et al., 2016; Shiue, 2013). The dispute remains existed between vitamin D and sleep disorders. Therefore, a meta‐analysis was performed evaluating the difference in vitamin D between sleep disorder people and normal people. Additionally, the meta‐analysis also evaluated all related randomized control trials (RCTs) with a focus on the influence of vitamin D supplementation on sleep disorders.

## 2 METHODS

### 2.1 Sources and methods of data retrieval

Literatures of PubMed, MEDLINE, Web of Science, and Cochrane Library were searched from these database inceptions until October 2019. We analyzed differences in vitamin D concentrations between people with sleep disorders and normal subjects. Meanwhile, sleep quality can be evaluated by the Pittsburgh Sleep Quality Index (PSQI) questionnaire, which consists of 19 items involving seven‐factor scores: subjective sleep quality, sleep latency, sleep duration, habitual sleep efficiency, sleep disturbances, use of sleeping pills, and daytime dysfunction. And higher scores indicate poorer sleep quality (Buysse, Reynolds, Monk, Berman, & Kupfer, 1989). Therefore, the change in the PSQI score in response to supplementation with vitamin D was also evaluated. The following terms were used for the literature search: vitamin D, cholecalciferol, ergocalciferol, sleep quality, sleep disorders, and Pittsburgh Sleep Quality Index (PSQI). The search strategy performed is detailed in Table 1. The location, assay method, sleep disorder types, study types, intervention dose and time of vitamin D, and other related factors were also evaluated.

**Table 1:** Search strategy

Criteria	Descriptions and search terms used for each criteria
Patient/population	(*sleep duration* OR *sleep quality* OR *sleep disorders* OR *short sleep* OR *hypersomnia* OR *sleep* OR *sleep time* OR *short‐term sleep restriction* OR *daytime sleepiness* OR *long sleepers* OR *short sleepers* OR *sleep initiation and maintenance disorders* OR *habitual short sleepers* OR *sleep deprivation* OR *nap* OR *napping* OR *sleep disturbance* OR *siesta* OR *sleep time* OR *drowse* OR *insomnia* OR *drowsiness* OR *24‐hr sleep duration* OR *night time sleep duration* OR *short sleep duration* OR *long sleep duration* OR *dyssomnia* OR *hypersomnia* OR *excessive sleepiness* OR *parasomnias*)
Exposure/Intervention	(*vitamin D analogues* OR *doxercalciferol* OR *alfacalcidol* OR *vitamin D3* OR *vitamin D2* OR *activated vitamin D* OR *1alpha‐vitamin D* OR *calcitriol* OR *calcidiol* OR *1,25dihydroxycholecalciferol* OR *25‐hydroxyvitamin D2* OR *calcifediol* OR *1,25OH2D* OR *dihydrotachysterol* OR *ergocalciferols* OR *25OHD* OR *Vit D* OR *25‐hydroxy vitamin D2* OR *VitD* OR *vitamin D−3* OR *25‐hydroxycholecalciferol* OR *25OHD* OR *25‐hydroxy‐vitamin D* OR *ergocalciferol* OR *1,25‐dihydroxyvitamin D3* OR *25‐OH vitamin D* OR *cholecalciferol* OR *25‐hydroxyvitamin D* OR *vitamin D*)

### 2.2 Inclusion criteria

The inclusion criteria were as follows: (a) Sleep disorder was defined on the basis of the standard. (b) The subjects had not taken vitamin D regularly previously. (c) We excluded patients who had hepatic or renal diseases, metabolic rickets, and any other medication that could influence vitamin D concentrations. (d) Studies that did not provide initial data, animal studies, duplicate literature, in vitro studies, reviews, or conference papers were excluded.

**Figure 1:**
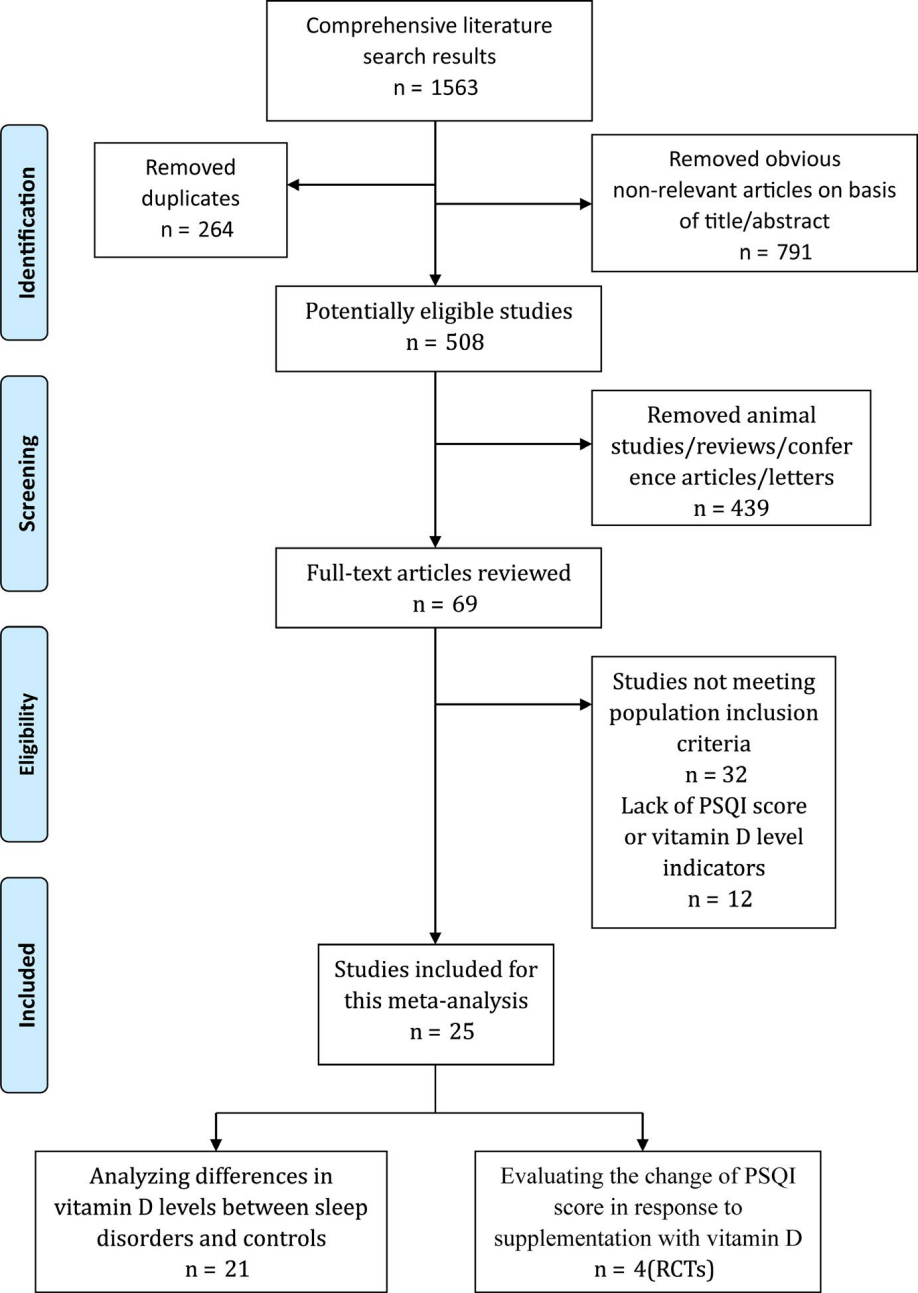
Flow diagram of the literature search and selection

The results must especially include quantitative data with specific values of vitamin D for the studies that analyze differences in vitamin D concentrations between people with sleep disorders and control subjects. Meanwhile, studies evaluating the sleep quality in response to supplementation with vitamin D must be RCTs, and the results must include specific values of the PSQI. Two researchers independently evaluated all studies, resolved divergence by discussion, and extracted final eligible literatures (Figure 1).

### 2.3 Data abstraction

All included literatures were assessed, and the following data were extracted: first author, nationality, publication year, numbers, mean age, and gender of case/supplementation groups and controls. We also extracted data on the assay method of vitamin D, sleep disorder types, and vitamin D levels in the subjects with sleep disorders and controls. Moreover, the intervention time and dose and the PSQI score in the vitamin D supplementation groups and controls were also extracted.

### 2.4 Risk of bias within individual studies

Cochrane Collaboration (RevMan version 5.3) software was used to estimate the risk of bias for RCTs. Simultaneously, the Newcastle–Ottawa scale (NOS) was used to estimate the risk of bias (including selection, comparability, and exposure) for the case–control study and cross‐sectional study.

### 2.5 Statistical analysis

Statistical analysis was performed using the statistical software RevMan version 5.3 and Stata version 12.0. The data from all the individual studies were used to calculate the standard mean difference (SMD) and 95% confidence intervals (CIs) using the random effects model. Cochran's Q statistic and the *I^2^* statistic were used to evaluate the statistical heterogeneity (Kochran, 1954). *p* < .05 was defined significant for heterogeneity (Higgins, Thompson, Deeks, & Altman, 2003). Heterogeneity was analyzed via sensitivity analysis and subgroup analyses. Egger's test was used to calculate the publication bias. Subgroup analyses were conducted based on the region (Eurasia (owing to Turkey's special geographical location), Europe and Asia), assay method of vitamin D (chemiluminescence immunoassay (CI), electrochemiluminescence immunoassay (EI), and other), sleep disorder type (obstructive sleep apnea syndrome (OSAS) (mild), OSAS (moderate), OSAS (severe), OSAS (unclassified), restless leg, and other), and study type (case–control study and cross‐sectional study) for the studies including subjects with sleep disorders and controls. Additionally, we used subgroup analyses based on the region (Asia and America), intervention time (≤2 months and >2 months), and serum vitamin D concentration after intervention (sufficiency (≥30 ng/ml) and insufficiency (<30 ng/ml)) to evaluate the source of heterogeneity for studies including the vitamin D supplementation groups and the controls.

## 3 RESULTS

Our study evaluated 1563 relevant literatures, but only 25 studies met the inclusion criteria, which contained a total of 3,603 subjects (Balaban et al., 2012; Bozkurt et al., 2012; Celik et al., 2015; Cikrikcioglu et al., 2016; Erden et al., 2014; Ghaderi et al., 2017; Gong et al., 2018; Gunduz et al., 2016; Han, Zhu, Shi, Wu, & Gu, 2017; Huang, Shah, Long, Crankshaw, & Tangpricha, 2013; Huzmeli, 2018; Kerley et al., 2016; Liguori et al., 2015; Majid et al., 2018; Mason et al., 2016; Mete et al., 2013; Neves et al., 2017; Pazarli, Ekiz, & Inonu Koseoglu, 2019; Qiao et al., 2018; Terzi & Yilmaz, 2016; Toujani et al., 2017; Uygur, Baki, Tanriverdi, Ornek, & Atalay, 2016; Wali et al., 2018; Zhao et al., 2017; Zicari et al., 2016). These 25 articles included 21 papers analyzing differences in vitamin D concentrations between sleep disorders and controls (Balaban et al., 2012; Bozkurt et al., 2012; Celik et al., 2015; Cikrikcioglu et al., 2016; Erden et al., 2014; Gong et al., 2018; Gunduz et al., 2016; Han et al., 2017; Huzmeli, 2018; Kerley et al., 2016; Liguori et al., 2015; Mete et al., 2013; Neves et al., 2017; Pazarli et al., 2019; Qiao et al., 2018; Terzi & Yilmaz, 2016; Toujani et al., 2017; Uygur et al., 2016; Wali et al., 2018; Zhao et al., 2017; Zicari et al., 2016) and 4 papers (RCTs) evaluating the change in the PSQI score in response to supplementation with vitamin D (Ghaderi et al., 2017; Huang et al., 2013; Majid et al., 2018; Mason et al., 2016). The detailed outcomes are presented in Table 2, Table 3, Table S1, and Table S2.

**Table 2:** Studies showing the serum vitamin D concentrations in patients with sleep disorder and controls

Author	Region	Year	Assay Method	Score	Sleep Disorder Types	*n*		Age		Vitamin D (ng/ml)		Gender (M/F)	
						Case	Control	Case	Control	Case	Control	Case	Control
Kerley et al (1).	Ireland	2015	CI^a^	8	OSAS^c^ (mild)	22	31	54.00 ± 19.00	53.00 ± 19.00	16.03 ± 8.81	24.04 ± 13.22	17/5	16/15
Kerley et al (2).	Ireland	2015	CI	8	OSAS (moderate)	18	31	57.00 ± 17.00	53.00 ± 19.00	14.94 ± 12.02	24.04 ± 13.22	12/6	16/15
Kerley et al (3).	Ireland	2015	CI	8	OSAS (severe)	35	31	55.50 ± 17.00	53.00 ± 19.00	14.82 ± 10.42	24.04 ± 13.22	28/7	16/15
Hatice et al (1).	Turkey	2012	EI^b^	8	restless leg	8	11	41.50 ± 6.27	36.45 ± 8.43	11.40 ± 6.23	12.99 ± 5.43	8/0	11/0
Hatice et al (2).	Turkey	2012	EI	8	restless leg	28	27	39.64 ± 7.65	37.96 ± 8.33	7.31 ± 4.63	12.31 ± 5.27	0/28	0/27
Cikrikcioglu et al.	Turkey	2016	EI	7	restless leg	78	78	46.45 ± 11.26	45.01 ± 12.27	14.18 ± 17.51	18.21 ± 18.25	0/78	0/78
Huzmeli et al.	Turkey	2018	‐	9	restless leg	33	42	59.60 ± 12.90	56.65 ± 15.77	10.76 ± 4.56	14.18 ± 7.02	9/24	26/16
Neves et al.	Brazil	2017	CI	8	restless leg	29	72	47.00 ± 18.00	45.00 ± 15.00	28.80 ± 10.20	30.50 ± 8.50	11/18	43/29
Celik et al.	Turkey	2015	EI	8	restless leg	31	31	43.61 ± 10.51	45.64 ± 14.43	15.10 ± 14.17	24.08 ± 22.47	0/31	0/31
Wali et al.	Saudi Arabia	2018	‐	8	restless leg	78	123	43.79 ± 6.04	44.75 ± 9.59	12.63 ± 7.03	26.07 ± 9.86	38/40	59/64
Claudio et al.	Italy	2015	‐	7	OSAS (severe)	90	32	61.10 ± 12.68	59.12 ± 8.02	19.34 ± 9.54	32.83 ± 16.93	60/24	22/10
Mete et al (1).	Turkey	2013	EI	7	OSAS (mild)	50	32	46.58 ± 9.37	46.94 ± 8.10	20.65 ± 9.65	19.02 ± 7.02	25/25	16/16
Mete et al (2).	Turkey	2013	EI	7	OSAS (moderate)	50	32	47.64 ± 7.22	46.94 ± 8.10	18.40 ± 9.02	19.02 ± 7.02	25/25	16/16
Mete et al (3).	Turkey	2013	EI	7	OSAS (severe)	50	32	47.40 ± 9.48	46.94 ± 8.10	14.66 ± 8.19	19.02 ± 7.02	25/25	16/16
Mete et al (4).	Turkey	2013	EI	7	OSAS (unclassified)	150	32	‐	46.94 ± 8.10	17.91 ± 9.25	19.02 ± 7.02	75/75	16/16
Zicari et al (1).	Italy	2016	CI	7	other	45	70	9.00 ± 1.75	9.04 ± 3.91	26.21 ± 10.70	34.07 ± 11.11	29/16	40/30
Zicari et al (2).	Italy	2016	CI	7	OSAS (unclassified)	22	70	7.62 ± 3.09	9.04 ± 3.91	20.80 ± 7.57	34.07 ± 11.11	15/7	40/30
Terzi et al.	Turkey	2015	other	8	other	30	20	52.37 ± 8.58	50.60 ± 10.84	14.06 ± 4.23	18.59 ± 6.68	30/0	20/0
Toujani et al.	Tunisia	2017	other	9	OSAS (severe)	92	30	52.30 ± 12.70	45.70 ± 14.70	7.90 ± 2.90	16.80 ± 3.10	48/44	17/13
Pazarli et al (1).	Turkey	2018	CI^a^	8	OSAS^c^ (mild)	28	21	46.50 ± 11.50	42.10 ± 1.80	17.15 ± 10.74	19.95 ± 15.87	17/11	10/11
Pazarli et al (2).	Turkey	2018	CI	8	OSAS (moderate)	13	21	51.00 ± 13.80	42.10 ± 1.80	17.19 ± 14.84	19.95 ± 15.87	5/8	10/11
Pazarli et al (3).	Turkey	2018	CI	8	OSAS (severe)	27	21	50.40 ± 11.90	42.10 ± 1.80	13.14 ± 10.58	19.95 ± 15.87	24/3	10/11
Gong et al.	China	2018	EI^b^	8	other	262	353	12.22 (1.75)	10.24 (1.73)	21.90 ± 5.70	24.30 ± 5.80	128/134	205/148
Uygur et al (1).	Turkey	2016	CI	6	OSAS (mild)	35	58	‐	‐	26.90 ± 8.40	31.00 ± 7.90	‐	‐
Uygur et al (2).	Turkey	2016	CI	6	OSAS (moderate)	35	58	‐	‐	22.30 ± 6.00	31.00 ± 7.90	‐	‐
Uygur et al (3).	Turkey	2016	CI	6	OSAS (severe)	33	58	‐	‐	17.60 ± 4.60	31.00 ± 7.90	‐	‐
Uygur et al (4).	Turkey	2016	CI	6	OSAS (unclassified)	103	58	46.70 ± 9.20	44.60 ± 9.70	22.40 ± 7.50	31.00 ± 7.90	54/49	23/35
Erden et al (1).	Turkey	2014	CI	9	OSAS (moderate)	23	43	42.00 ± 9.00	45.00 ± 14.00	22.05 ± 7.19	29.54 ± 9.09	17/6	21/22
Erden et al (2).	Turkey	2014	CI	9	OSAS (severe)	62	43	51.00 ± 10.00	45.00 ± 14.00	23.53 ± 7.73	29.54 ± 9.09	53/9	21/22
Erden et al (3).	Turkey	2014	CI	9	OSAS (unclassified)	85	43	48.56 ± 9.74	45.00 ± 14.00	23.13 ± 7.57	29.54 ± 9.09	70/15	21/22
Zhao et al.	China	2017	EI	8	other	181	100	43.16 ± 10.78	44.31 ± 10.33	23.01 ± 9.18	27.17 ± 6.41	52/129	32/68
Gunduz et al.	Turkey	2016	other	8	other	58	34	29.70 ± 4.80	30.50 ± 4.20	22.10 ± 16.40	24.30 ± 16.10	0/58	0/34
Han et al.	China	2017	‐	7	other	88	53	59.70 ± 15.30	62.80 ± 12.50	15.64 ± 11.64	34.24 ± 14.96	52/36	34/19
Bozkurt et al (1).	Turkey	2012	other	8	OSAS (severe)	50	47	49.66 ± 10.38	42.79 ± 9.55	16.31 ± 6.98	19.93 ± 7.81	29/21	28/19
Bozkurt et al (2).	Turkey	2012	other	8	OSAS (moderate)	47	47	49.79 ± 10.62	42.79 ± 9.55	17.55 ± 7.42	19.93 ± 7.81	28/19	28/19
Bozkurt et al (3).	Turkey	2012	other	8	OSAS (mild)	46	47	47.78 ± 10.35	42.79 ± 9.55	18.29 ± 6.48	19.93 ± 7.81	28/18	28/19
Bozkurt et al (4).	Turkey	2012	other	8	OSAS (unclassified)	143	47	‐	42.79 ± 9.55	17.40 ± 6.90	19.93 ± 7.81	85/58	28/19
Qiao et al (1).	China	2018	EI	8	OSAS (moderate)	32	32	51.80 ± 8.10	50.10 ± 7.30	17.62 ± 5.88	27.23 ± 7.59	32/0	32/0
Qiao et al (2).	China	2018	EI	8	OSAS (severe)	55	32	48.20 ± 9.90	50.10 ± 7.30	10.83 ± 6.80	27.23 ± 7.59	55/0	32/0

^a^ Chemiluminescence immunoassay.

^b^ Electrochemiluminescence immunoassay.

^c^ Obstructive sleep apnea syndrome.

**Table 3:** Studies showing the PSQI score in vitamin D supplementation groups and control groups

Author	Region	Year	Intervention Dose	Intervention Time	*n*		Age		Gender (M/F)		PSQI score	
					Study	Control	Supplementation Groups	Control	Supplementation Groups	Control	Supplementation Groups	Control
Majid et al (1).	Iran	2017	25000IU/week	8 weeks	44	45	37.90 ± 9.50	35.50 ± 10.00	11/33	10/35	6.75 ± 2.97	9.73 ± 3.04
Majid et al (2).	Iran	2017	25000IU/week	8 weeks	44	44	37.90 ± 9.50	37.90 ± 9.50	11/33	11/33	6.75 ± 2.97	9.45 ± 2.44
Ghaderi et al (1).	Iran	2017	25000IU/week	12 weeks	34	34	40.10 ± 9.20	42.50 ± 8.90	—	—	4.50 ± 2.20	6.40 ± 3.00
Ghaderi et al (2).	Iran	2017	25000IU/week	12 weeks	34	34	40.10 ± 9.20	40.10 ± 9.20	—	—	4.50 ± 2.20	6.00 ± 2.30
Wei et al (1).	the U.S.	2013	‐	3 months	28	28	46.20 ± 10.80	46.20 ± 10.80	18/10	18/10	12.22 ± 4.61	13.46 ± 4.92
Wei et al (2).	the U.S.	2013	1,200 IU/day	3 months	15	15	47.55 ± 12.00	47.55 ± 12.00	11/4	11/4	11.29 ± 4.66	12.27 ± 5.55
Wei et al (3).	the U.S.	2013	50,000 IU/week	3 months	13	13	44.58 ± 9.46	44.58 ± 9.46	7/6	7/6	13.23 ± 4.51	14.85 ± 3.83
Mason et al (1).	the U.S.	2016	2000 IU/day	12 months	83	84	—	—	0/83	0/84	7.60 ± 2.90	7.40 ± 2.80
Mason et al (2).	the U.S.	2016	2000 IU/day	12 months	83	103	—	—	0/83	0/103	7.60 ± 2.90	7.50 ± 2.90

### 3.1 Differences in vitamin D concentrations between sleep disorders and controls

For the 21 papers analyzing differences in vitamin D levels, 11 studies were conducted in Eurasia (Balaban et al., 2012; Bozkurt et al., 2012; Celik et al., 2015; Cikrikcioglu et al., 2016; Erden et al., 2014; Gunduz et al., 2016; Huzmeli, 2018; Mete et al., 2013; Pazarli et al., 2019; Terzi & Yilmaz, 2016; Uygur et al., 2016), 3 studies were performed in Europe (Kerley et al., 2016; Liguori et al., 2015; Zicari et al., 2016), and 5 studies were performed in Asia (Gong et al., 2018; Han et al., 2017; Qiao et al., 2018; Wali et al., 2018; Zhao et al., 2017) (the America group and Africa group were not analyzed because only 1 paper was included, respectively (Neves et al., 2017; Toujani et al., 2017)). Seven studies used EI to analyze the vitamin D levels (Balaban et al., 2012; Celik et al., 2015; Cikrikcioglu et al., 2016; Gong et al., 2018; Mete et al., 2013; Qiao et al., 2018; Zhao et al., 2017), and 6 studies used CI (Erden et al., 2014; Kerley et al., 2016; Neves et al., 2017; Pazarli et al., 2019; Uygur et al., 2016; Zicari et al., 2016), while the remaining studies (Bozkurt et al., 2012; Gunduz et al., 2016; Terzi & Yilmaz, 2016; Toujani et al., 2017) (*n* = 4)used other methods (4 studies (Han et al., 2017; Huzmeli, 2018; Liguori et al., 2015; Wali et al., 2018) did not provide an assay method). The type of sleep disorder was OSAS (mild) in 5 studies (Bozkurt et al., 2012; Kerley et al., 2016; Mete et al., 2013; Pazarli et al., 2019; Uygur et al., 2016), OSAS (moderate) in 7 studies (Bozkurt et al., 2012; Erden et al., 2014; Kerley et al., 2016; Mete et al., 2013; Pazarli et al., 2019; Qiao et al., 2018; Uygur et al., 2016), OSAS (severe) in 9 studies (Bozkurt et al., 2012; Erden et al., 2014; Kerley et al., 2016; Liguori et al., 2015; Mete et al., 2013; Pazarli et al., 2019; Qiao et al., 2018; Toujani et al., 2017; Uygur et al., 2016), OSAS (unclassified) in 5 studies (Bozkurt et al., 2012; Erden et al., 2014; Mete et al., 2013; Uygur et al., 2016; Zicari et al., 2016), restless legs syndrome in 6 studies (Balaban et al., 2012; Celik et al., 2015; Cikrikcioglu et al., 2016; Huzmeli, 2018; Neves et al., 2017; Wali et al., 2018), and other in 6 studies (Gong et al., 2018; Gunduz et al., 2016; Han et al., 2017; Terzi & Yilmaz, 2016; Zhao et al., 2017; Zicari et al., 2016). Seventeen studies were case–control studies (Balaban et al., 2012; Bozkurt et al., 2012; Celik et al., 2015; Cikrikcioglu et al., 2016; Erden et al., 2014; Han et al., 2017; Huzmeli, 2018; Liguori et al., 2015; Mete et al., 2013; Neves et al., 2017; Qiao et al., 2018; Terzi & Yilmaz, 2016; Toujani et al., 2017; Uygur et al., 2016; Wali et al., 2018; Zhao et al., 2017; Zicari et al., 2016), and 4 studies were cross‐sectional studies (Gong et al., 2018; Gunduz et al., 2016; Kerley et al., 2016; Pazarli et al., 2019). The risk of bias within individual studies for analyzing differences in vitamin D levels via NOS is presented in Table 2 and Table S1. In addition, the GRADE system was conducted to determine the quality of evidence (Table 4).

**Table 4:** The Summary of Findings (SoF) with GRADE system (vitamin D levels)

Good sleep quality compared with poor sleep quality in vitamin D levels			
Population: Subjects with sleep disorders vs. normal subjects Settings: Eleven studies were conducted in Eurasia; three studies were conducted in Europe; five studies were conducted in Asia; one study was conducted in America; one study was conducted in Africa. Cases: Subjects with sleep disorders Controls: Normal subjects			
**Outcomes**	**SMD (95% CI)** ^a^	**No of participants (studies)**	**Quality of the evidence Comments (GRADE)**
Vitamin D levels	−0.75 (−0.93, −0.57)	3204 (21 case–control/cross‐sectional studies)	⊕⊕⊕⊕HIGH^b^, ^c^

GRADE working group grades of evidence.

High quality: We are very confident that the true effect lies close to that of the estimate of the effect.

Moderate quality: We are moderately confident in the effect estimate: The true effect is likely to be close to the estimate of the effect, but there is a possibility that it is substantially different.

Low quality: Our confidence in the effect estimate is limited: The true effect may be substantially different from the estimate of the effect.

Very low quality: We have very little confidence in the effect estimate: The true effect is likely to be substantially different from the estimate of effect.

Abbreviations: CI, confidence interval; SMD, standard mean deviation.

^a^ Results for vitamin D levels of subjects with sleep disorders compared with controls.

^b^ Upgraded by one level due to all the results of the included studies were almost identical(subjects with sleep disorders had lower vitamin D levels).

^c^ Upgraded by one level due to sleep disorders were associated with vitamin D levels(The more serious the sleep disorder, the lower the vitamin D levels).

**Figure 2:**
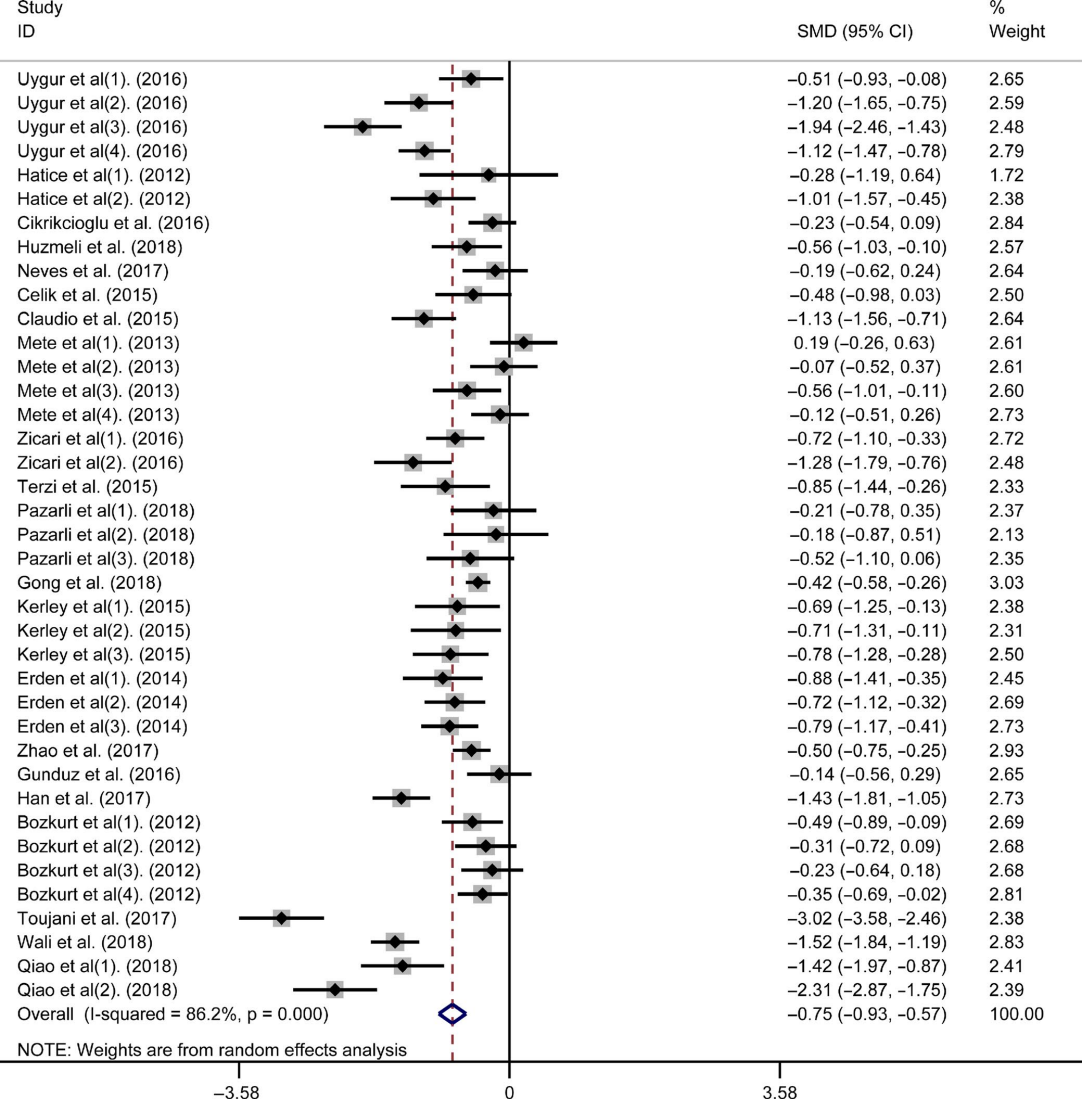
Forest plot of the vitamin D concentration in the sleep disorders vs. control groups

We performed a meta‐analysis of the serum vitamin D concentration in 1864 sleep disorder subjects and 1,340 control peoples. The average serum vitamin D concentration in the sleep disorder subjects was 0.75ng/ml lower than that in the control group (SMD = −0.75 ng/ml, 95% CI = −0.93,−0.57 ng/ml, *I^2^* = 86.2%, *p < *.001; Figure 2). Simultaneously, publication bias was not found in the serum vitamin D concentration (Egger's test: coefficient = −0.255, *t *= −0.98, *p* = .334). Additionally, the subgroup analysis was conducted based on the region, assay method of vitamin D, sleep disorder types, and study types. The details are shown in Table 5. Studies were separated into three groups: Eurasia, Asia, and Europe based on the geographical study area. For the three groups, the average serum vitamin D concentrations were lower than those of the controls (Figure S1). Simultaneously, a subgroup analysis was conducted based on the assay method, and the studies were separated into three methods: CI, EI, and other. The average serum vitamin D concentrations were lower than those of the controls in all three groups (Figure S2). The main types of sleep disorders in the included studies were OSAS (mild), OSAS (moderate), OSAS (severe), OSAS (unclassified), restless legs syndrome, and other. The average serum vitamin D concentrations were lower in all of the groups compared to those of the controls except for the OSAS (mild) group (Figure S3). Moreover, we separated the studies into two groups (case–control study and cross‐sectional study) based on the included study types. For the two groups, statistically significant differences with the controls were observed (Figure S4).

**Table 5:** Subgroup analyses for the serum vitamin D concentrations in patients with sleep disorder and controls

Subgrouped by	No. of studies	SMD	95% CI	*I^2^* (%)	*P* for heterogeneity
Region					
Eurasia	11	−0.54	−0.72, −0.37	73.1	＜.001
Europe	3	−0.89	−1.09, −0.69	3.8	.392
Asia	5	−1.23	−1.77, −0.69	94.4	＜.001
Assay method					
EI	7	−0.58	−0.86, −0.29	85.2	＜.001
CI	6	−0.79	−1.00, −0.58	68.1	＜.001
Other methods	4	−0.75	−1.36, −0.14	92.8	＜.001
Sleep disorder types					
OSAS (mild)	5	−0.27	−0.56, 0.01	46.4	.113
OSAS (moderate)	7	−0.68	−1.08, −0.29	75.8	＜.001
OSAS (severe)	9	−1.26	−1.82, −0.71	91.7	＜.001
OSAS (unclassified)	5	−0.72	−1.14, −0.30	83.1	＜.001
Restless leg	6	−0.63	−1.08, −0.17	85.7	＜.001
Other types	6	−0.66	−0.97, −0.34	82.7	＜.001
Study types					
Case–control study	17	−0.83	−1.05, −0.61	88.0	＜.001
Cross‐sectional study	4	−0.43	−0.56, −0.30	0.0	.473

### 3.2 Effect of vitamin D supplementation on sleep disorders

For the 4 papers evaluating the change in the PSQI score, 2 studies were performed in Asia (Ghaderi et al., 2017; Majid et al., 2018), and the remaining studies (Huang et al., 2013; Mason et al., 2016) (*n* = 2) were conducted in America. The intervention time was ≤2 months in 1 paper (Majid et al., 2018) and >2 months in 3 studies (Ghaderi et al., 2017; Huang et al., 2013; Mason et al., 2016). The serum vitamin D concentrations after intervention were sufficient (Majid et al., 2018; Mason et al., 2016) in 2 papers and insufficient in the others (Ghaderi et al., 2017; Huang et al., 2013). The basic situation of the subjects is shown in Table 3 and Table S2.

**Figure 3:**
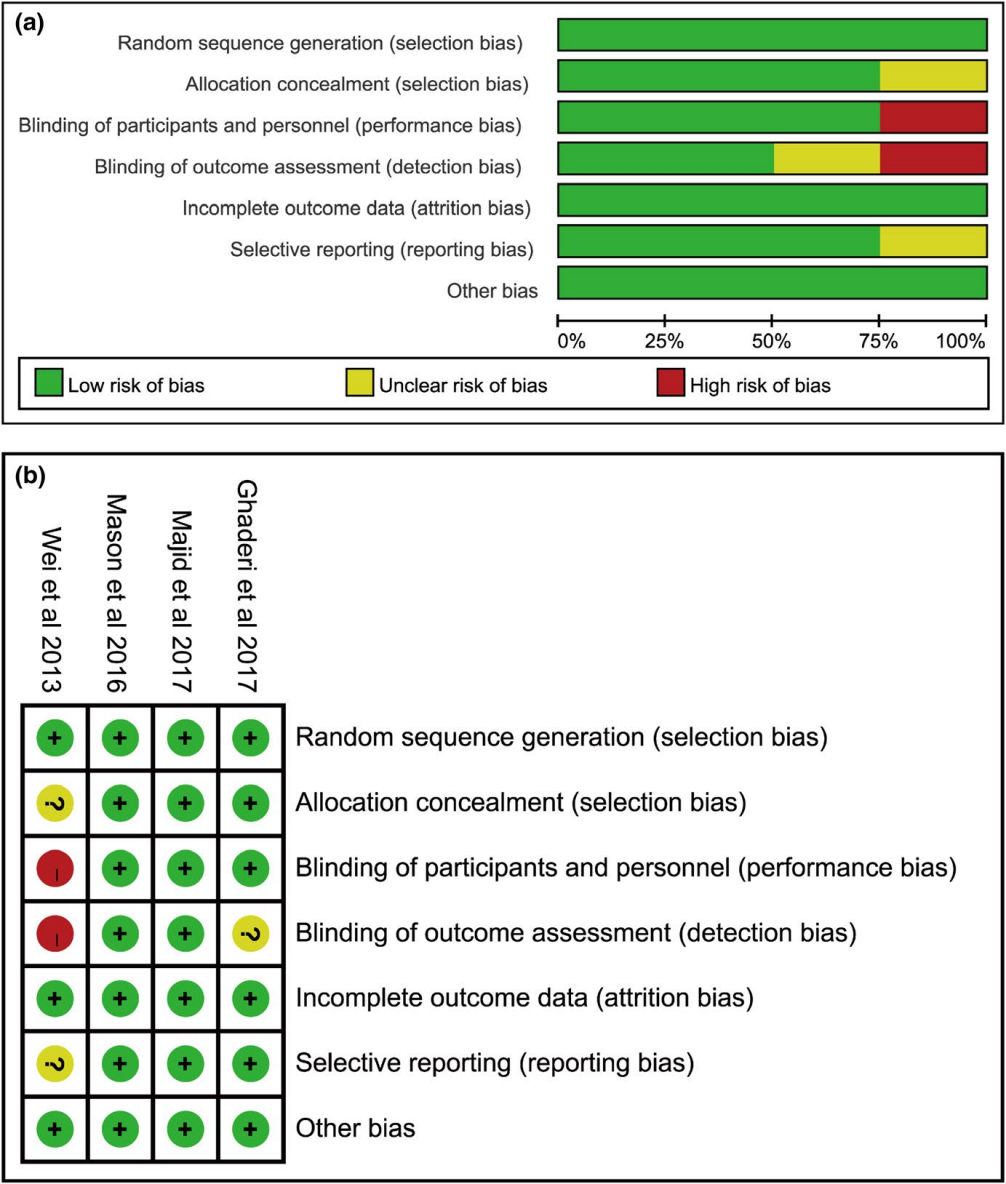
Risk of within‐study bias (RCT)

The risk of bias within individual studies for evaluating the change in the PSQI score is shown in Figure 3 and Table S2. All 4 studies were randomized and had complete outcome data (Ghaderi et al., 2017; Huang et al., 2013; Majid et al., 2018; Mason et al., 2016). Additionally, 3 trials might have controlled the reporting bias via registering in a clinical trial registry (Ghaderi et al., 2017; Majid et al., 2018; Mason et al., 2016). The methods of allocation concealment and blinding of participants and study personnel were properly described in 3 studies (Ghaderi et al., 2017; Majid et al., 2018; Mason et al., 2016). Two studies conducted the methods of blinding of the outcome (Majid et al., 2018; Mason et al., 2016). Moreover, no commercial company was involved and no conflict of interest existed in all the studies, so the studies were considered free of potential bias (Ghaderi et al., 2017; Huang et al., 2013; Majid et al., 2018; Mason et al., 2016). Similarly, the GRADE system was conducted to determine the quality of evidence (Table 6).

**Table 6:** The Summary of Findings (SoF) with GRADE system (PSQI score)

Vitamin D supplementation compared with no vitamin D intervention for improving sleep quality			
Population: Subjects with sleep disorders Settings: Two studies were conducted in Asia, and two studies were conducted in America Intervention: Vitamin D supplementation Comparison: No vitamin D intervention			
**Outcomes** ^a^	**SMD (95% CI)** ^b^	**No of participants (studies)**	**Quality of the evidence Comments (GRADE)**
PSQI score	−0.45 (−0.76, −0.13)	399 (4RCTs)	⊕⊕⊕⊕HIGH

GRADE Working group grades of evidence.

High quality: We are very confident that the true effect lies close to that of the estimate of the effect.

Moderate quality: We are moderately confident in the effect estimate: The true effect is likely to be close to the estimate of the effect, but there is a possibility that it is substantially different.

Low quality: Our confidence in the effect estimate is limited: The true effect may be substantially different from the estimate of the effect.

Very low quality: We have very little confidence in the effect estimate: The true effect is likely to be substantially different from the estimate of effect.

Abbreviations: CI, confidence interval; RCT, randomized controlled trial; SMD, standard mean deviation.

^a^ All subjects were followed up range 8 weeks to 12 months.

^b^ Results for PSQI score of treatments compared with controls (including PSQI score of postsupplementation compared with presupplementation in the treatments).

**Figure 4:**
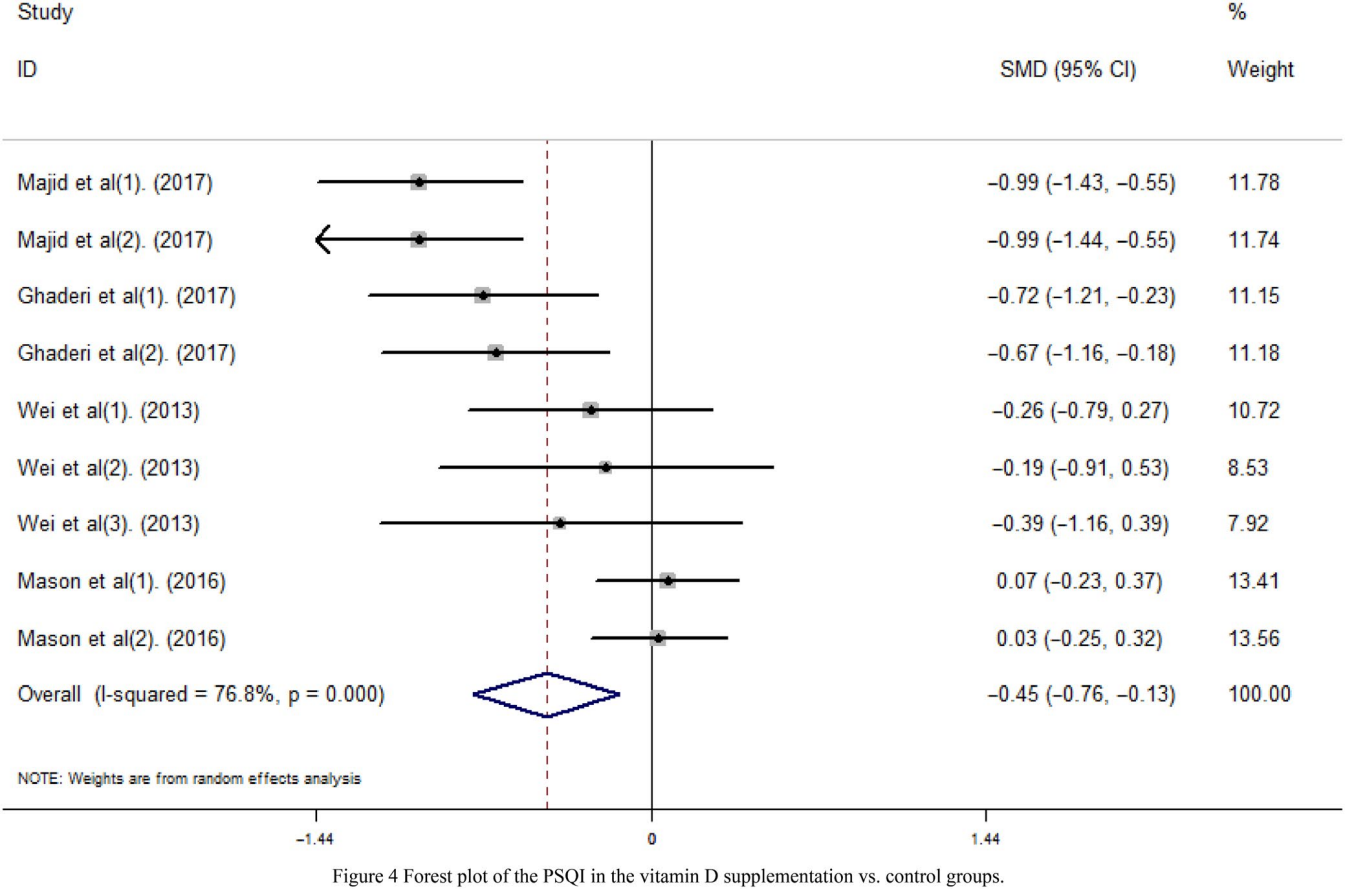
Forest plot of the PSQI in the vitamin D supplementation vs. control groups

We performed a meta‐analysis of the PSQI in 189 subjects with vitamin D supplementation and 210 control subjects. The PSQI in the vitamin D supplementation group was 0.45 lower than that in the control group (SMD = −0.45, 95% CI = −0.76, −0.13, *I^2^* = 76.8%, *p* < .001; Figure 4). Similarly, publication bias was not found in the PSQI (Egger's test: coefficient = −3.29, *t *= −1.56, *p* = .164). Moreover, the subgroup analysis was conducted based on the region, intervention time, and serum vitamin D concentration after intervention. Details are shown in Table 7. Studies were separated into two regions based on the geographical study area. For the Asia group, the PSQI was lower than that of the controls. However, the researches in America did not indicate differences in the PSQI between the supplementation subjects and control subjects (Figure S5). Simultaneously, the studies were separated into two groups based on the intervention time: ≤2 months and >2 months. The PSQI was lower than that of the controls in the ≤2 months' group, while the studies in the >2 months' group did not indicate differences between the supplementation and control subjects (Figure S6). Additionally, all subjects were separated into two groups based on their serum vitamin D levels after intervention (Figure S7).

**Table 7:** Subgroup analyses for the PSQI score in vitamin D supplementation groups and control groups

Subgrouped by	No. of studies	SMD	95% CI	*I^2^* (%)	*P* for heterogeneity
Region					
Asia	2	−0.86	−1.09, −0.63	0.0	.661
America	2	−0.03	−0.21, 0.16	0.0	.672
Intervention time					
≤2 months	1	−0.99	−1.31, −0.68	0.0	.995
＞2 months	3	−0.26	−0.52, 0.00	55.8	.035
Vitamin D levels after intervention					
Sufficiency	2	−0.45	−1.01, 0.12	89.9	＜.001
Insufficiency	2	−0.50	−0.75, −0.24	0.0	.588

## 4 DISCUSSION

Sleep disorders bring a heavy burden on the healthcare system. The personal average annual medical expense may increase by $2000 due to chronic sleep disorders (Xie et al., 2017). It has been reported that vitamin D deficiency is related to a higher risk of sleep disorders (Gao et al., 2018). However, it is controversial whether supplementation with vitamin D is truly beneficial to improving sleep quality. This meta‐analysis found that vitamin D concentrations in sleep disorders were significantly lower than those in normal controls. In addition, vitamin D supplementation can effectively improve sleep quality. Although the specific mechanism of the role of vitamin D on sleep disorders has not yet been illustrated, some potential mechanisms have been considered. Low concentrations of vitamin D can disrupt sleep by creating and developing myopathic pain (Lee, Greenfield, & Campbell, 2009). Meanwhile, as central sleep regulators, inflammatory mediators (including TNF‐α and IL‐1) and prostaglandin D2 indicated negative correlation with vitamin D levels. Inflammatory mediators and prostaglandin D2 were increased in cases of vitamin D deficiency, thus leading to sleep disorders, including OSAS. Therefore, vitamin D supplementation could effectively improve sleep quality (Barcelo et al., 2007; Bellia et al., 2013; Khoo et al., 2011).

When we analyzed the outcomes of the vitamin D concentrations and the PSQI score in the included papers, a high level of heterogeneity was found in both outcomes, so we conducted a subgroup analysis to determine the source of heterogeneity. The subgroup analysis of the region indicated that the heterogeneity was decreased in both results. Therefore, we concluded that different regions could be the source of heterogeneity in the included studies. In addition, the PSQI was significantly lower in the vitamin D supplementation subjects than in the control subjects for the Asia group, while there was no significant difference for the America group. Several decades ago, as one of the industrialized countries, America had undertaken fortification of milk and other food products with vitamin D (Marwaha & Dabas, 2019). Therefore, there may be other factors or diseases that cause sleep disorders in Americans. In contrast, in Asian countries, urbanization is likely to be related to lifestyle changes, lower physical activity, an increase in indoor living, and lack of sun exposure, thus leading to an increase in vitamin D deficiency (Mithal, Bansal, Kyer, & Ebeling, 2014). Additionally, in different areas of Asia, nutritional status and sunlight exposure are diversity; hence, the vitamin D supplementation may be more necessary for Asian people (Lau et al., 2006). Furthermore, due to Turkey's special geographical location (across Eurasia), we took it as a subgroup when we analyzed the differences in vitamin D concentrations between the sleep disorders and control subjects by subgroup analysis based on region. We considered the combination of east and west in various fields, and the complexity of race may be the reason for the high heterogeneity in the Eurasia group.

Comparing the subjects with sleep disorders with the control group at the vitamin D level, the results indicated that OSAS severity may correlate with vitamin D levels. As one of the major sleep disorders, the more serious the disease is, the lower the level of vitamin D. The result is similar to the K's study (Archontogeorgis, Nena, Papanas, Zissimopoulos, et al., 2018). Although the mechanism of vitamin D insufficiency or deficiency in OSAS is inadequately understood, several possible pathogeneses indicated that they may affect each other. OSAS subjects are probably to have excessive daytime sleepiness or obesity, thus reducing the outdoor activities and sunlight exposure, leading to the decrease in vitamin D synthesis (Igelstrom, Emtner, Lindberg, & Asenlof, 2013). Meanwhile, vitamin D has immunomodulatory properties. Multiple immune cells, such as antigen‐presenting cells, T cells, B cells, and monocytes, have vitamin D metabolizing enzymes and vitamin D receptors (Archontogeorgis, Nena, Papanas, & Steiropoulos, 2018; Prietl, Treiber, Pieber, & Amrein, 2013). Recurrent infections and immune system imbalance caused by vitamin D deficiency could lead to tonsillar hypertrophy and chronic rhinitis, both of which elevate the risk of OSAS or aggravate it (Reid, Morton, Salkeld, & Bartley, 2011). Therefore, OSAS patients are more likely to fall into the vicious circle of vitamin D deficiency‐OSAS aggravation.

According to the results of the vitamin D supplementation, the studies in >2 months did not demonstrate differences between the supplementation and control subjects. The reason may be that the guidelines for vitamin D supplementation in people with sleep disorders were lacking. We could only refer to the existing supplemental guidelines, which suggested 50,000 IU once a week for 8 weeks for clinical management of vitamin D deficiency in adults (Cesareo et al., 2018) (since only one group in the included studies was 50,000 IU/week, we were unable to perform the subgroup analysis according to intervention dose). Therefore, considering the small sample size, more RCTs are required to assess the relationship between vitamin D supplementation and sleep disorders. In the meantime, a guideline of vitamin D supplementation for sleep disorders patients is urgently needed.

This meta‐analysis also has some limitations. Some papers did not provide the detection method of vitamin D, so we could not include it when the subgroup analysis was conducted based on the detection method. Meanwhile, few studies met the existing guidelines for vitamin D supplementation, so we could not conduct a subgroup analysis based on the intervention dose. Most importantly, there are fewer RCTs about vitamin D supplementation for sleep disorders. Although RCTs are supposed to valid evidence compared to other studies, true vitamin D supplementation roles could be biased by the quality of the data from the original papers and the limited sample size and included studies.

## 5 CONCLUSIONS

Vitamin D could play a promising role in sleep disorders. Considering several limitations found in this meta‐analysis, more data from RCTs are required to confirm the efficacy of vitamin D supplementation for improving sleep disorders.

## CONFLICT OF INTEREST

The authors declare that they have no competing interests.

## AUTHORS' CONTRIBUTIONS

BL, WC, and SY made the study design; SY, ZT, HZ, and YP conducted the study; SY, ZT, CW, and NY analyzed the data and wrote the manuscript; SY, ZT, YG, and HW participated amending the manuscript. SY and ZT contributed equally to this work. All authors agreed with the final version of the manuscript.

## STUDIES INVOLVING HUMAN SUBJECTS

Although the study involves human subjects, it is a meta‐analysis based on evaluating published research data. Therefore, no ethical issues are involved.

## Supporting information

FigS1

FigS2

FigS3

FigS4

FigS5

FigS6

FigS7

TableS1

TableS2

## References

[fsn31867-bib-0001] Archontogeorgis, K. , Nena, E. , Papanas, N. , & Steiropoulos, P. (2018). The role of vitamin D in obstructive sleep apnoea syndrome. Breathe (Sheff), 14 (3), 206–215. 10.1183/20734735.000618 30186518 PMC6118887

[fsn31867-bib-0002] Archontogeorgis, K. , Nena, E. , Papanas, N. , Zissimopoulos, A. , Voulgaris, A. , Xanthoudaki, M. , … Steiropoulos, P. (2018). Vitamin D levels in middle‐aged patients with obstructive sleep apnoea syndrome. Current Vascular Pharmacology, 16 (3), 289–297. 10.2174/1570161115666170529085708 28552071

[fsn31867-bib-0003] Balaban, H. , Yildiz, O. K. , Cil, G. , Senturk, I. A. , Erselcan, T. , Bolayir, E. , & Topaktas, S. (2012). Serum 25‐hydroxyvitamin D levels in restless legs syndrome patients. Sleep Medicine, 13 (7), 953–957. 10.1016/j.sleep.2012.04.009 22704399

[fsn31867-bib-0004] Barcelo, A. , de la Pena, M. , Barbe, F. , Pierola, J. , Bosch, M. , & Agusti, A. G. (2007). Prostaglandin D synthase (beta trace) levels in sleep apnea patients with and without sleepiness. Sleep Medicine, 8 (5), 509–511. 10.1016/j.sleep.2006.10.005 17512779

[fsn31867-bib-0005] Bellia, A. , Garcovich, C. , D’Adamo, M. , Lombardo, M. , Tesauro, M. , Donadel, G. , … Sbraccia, P. (2013). Serum 25‐hydroxyvitamin D levels are inversely associated with systemic inflammation in severe obese subjects. Internal and Emergency Medicine, 8 (1), 33–40. 10.1007/s11739-011-0559-x 21437585

[fsn31867-bib-0006] Bozkurt, N. C. , Cakal, E. , Sahin, M. , Ozkaya, E. C. , Firat, H. , & Delibasi, T. (2012). The relation of serum 25‐hydroxyvitamin‐D levels with severity of obstructive sleep apnea and glucose metabolism abnormalities. Endocrine, 41 (3), 518–525. 10.1007/s12020-012-9595-1 22246808

[fsn31867-bib-0007] Buysse, D. J. , Reynolds, C. F. 3rd , Monk, T. H. , Berman, S. R. , & Kupfer, D. J. (1989). The Pittsburgh Sleep Quality Index: A new instrument for psychiatric practice and research. Psychiatry Research, 28 (2), 193–213. 10.1016/0165-1781(89)90047-4 2748771

[fsn31867-bib-0008] Celik, K. , Cikrikcioglu, M. A. , Halac, G. U. , Kilic, E. , Ayhan, S. , Ozaras, N. , … Kiskac, M. (2015). Serum endocan levels in women with restless legs syndrome. Neuropsychiatric Disease and Treatment, 11, 2919–2925. 10.2147/ndt.s92771 26640378 PMC4657799

[fsn31867-bib-0009] Cesareo, R. , Attanasio, R. , Caputo, M. , Castello, R. , Chiodini, I. , Falchetti, A. , … Zini, M. (2018). Italian Association of Clinical Endocrinologists (AME) and Italian Chapter of the American Association of Clinical Endocrinologists (AACE) Position Statement: Clinical Management of Vitamin D Deficiency in Adults. Nutrients, 10 (5), 546 10.3390/nu10050546 PMC5986426 29702603

[fsn31867-bib-0010] Cikrikcioglu, M. A. , Sekin, Y. , Halac, G. , Kilic, E. , Kesgin, S. , Aydin, S. , … Kiskac, M. (2016). Reduced bone resorption and increased bone mineral density in women with restless legs syndrome. Neurology, 86 (13), 1235–1241. 10.1212/wnl.0000000000002521 26920357

[fsn31867-bib-0011] de Oliveira, D. L. , Hirotsu, C. , Tufik, S. , & Andersen, M. L. (2017). The interfaces between vitamin D, sleep and pain. Journal of Endocrinology, 234 (1), R23–R36. 10.1530/joe-16-0514 28536294

[fsn31867-bib-0012] Erden, E. S. , Genc, S. , Motor, S. , Ustun, I. , Ulutas, K. T. , Bilgic, H. K. , … Gokce, C. (2014). Investigation of serum bisphenol A, vitamin D, and parathyroid hormone levels in patients with obstructive sleep apnea syndrome. Endocrine, 45 (2), 311–318. 10.1007/s12020-013-0022-z 23904340

[fsn31867-bib-0013] Gao, Q. , Kou, T. , Zhuang, B. , Ren, Y. , Dong, X. , & Wang, Q. (2018). The Association between vitamin D deficiency and sleep disorders: A systematic review and meta‐analysis. Nutrients, 10 (10), 10.3390/nu10101395 PMC6213953 30275418

[fsn31867-bib-0014] Gaultney, J. F. (2010). The prevalence of sleep disorders in college students: Impact on academic performance. Journal of American College Health, 59 (2), 91–97. 10.1080/07448481.2010.483708 20864434

[fsn31867-bib-0015] Ghaderi, A. , Banafshe, H. R. , Motmaen, M. , Rasouli‐Azad, M. , Bahmani, F. , & Asemi, Z. (2017). Clinical trial of the effects of vitamin D supplementation on psychological symptoms and metabolic profiles in maintenance methadone treatment patients. Progress in Neuro‐Psychopharmacology and Biological Psychiatry, 79 (Pt B), 84–89. 10.1016/j.pnpbp.2017.06.016 28642082

[fsn31867-bib-0016] Gominak, S. C. , & Stumpf, W. E. (2012). The world epidemic of sleep disorders is linked to vitamin D deficiency. Medical Hypotheses, 79 (2), 132–135. 10.1016/j.mehy.2012.03.031 22583560

[fsn31867-bib-0017] Gong, Q. H. , Li, S. X. , Li, H. , Chen, Q. , Li, X. Y. , & Xu, G. Z. (2018). 25‐Hydroxyvitamin D Status and Its Association with Sleep Duration in Chinese Schoolchildren. Nutrients, 10 (8), 10.3390/nu10081013 PMC6116160 30081486

[fsn31867-bib-0018] Gunduz, S. , Kosger, H. , Aldemir, S. , Akcal, B. , Tevrizci, H. , Hizli, D. , & Celik, H. T. (2016). Sleep deprivation in the last trimester of pregnancy and inadequate vitamin D: Is there a relationship? Journal of the Chinese Medical Association, 79 (1), 34–38. 10.1016/j.jcma.2015.06.017 26391786

[fsn31867-bib-0019] Han, B. , Zhu, F. X. , Shi, C. , Wu, H. L. , & Gu, X. H. (2017). Association between serum vitamin D levels and sleep disturbance in hemodialysis patients. Nutrients, 9 (2), 10.3390/nu9020139 PMC5331570 28216568

[fsn31867-bib-0020] Higgins, P. T. , Thompson, S. G. , Deeks, J. J. , & Altman, D. G. ( 2003). Measuring inconsistency in meta‐analyses. BMJ, 327 (7414), 557–560. 10.1136/bmj.327.7414.557 12958120 PMC192859

[fsn31867-bib-0021] Huang, W. , Shah, S. , Long, Q. , Crankshaw, A. K. , & Tangpricha, V. (2013). Improvement of pain, sleep, and quality of life in chronic pain patients with vitamin D supplementation. Clinical Journal of Pain, 29 (4), 341–347. 10.1097/AJP.0b013e318255655d 22699141

[fsn31867-bib-0022] Huzmeli, C. (2018). The relationship between restless leg syndrome and 25‐hydroxy d vitamin in hemodialysis patients. Journal of Clinical and Analytical Medicine, 9 (1), 10.4328/JCAM.5452

[fsn31867-bib-0023] Igelstrom, H. , Emtner, M. , Lindberg, E. , & Asenlof, P. (2013). Physical activity and sedentary time in persons with obstructive sleep apnea and overweight enrolled in a randomized controlled trial for enhanced physical activity and healthy eating. Sleep Breath, 17 (4), 1257–1266. 10.1007/s11325-013-0831-6 23536259

[fsn31867-bib-0024] Institute of Medicine Committee on Sleep, M., & Research . (2006). The National Academies Collection: Reports funded by National Institutes of Health In Colten H. R., & Altevogt B. M. (Eds.), Sleep Disorders and Sleep Deprivation: An Unmet Public Health Problem. Washington (DC): National Academies Press (US), National Academy of Sciences. 20669438

[fsn31867-bib-0025] Kerley, C. P. , Hutchinson, K. , Bolger, K. , McGowan, A. , Faul, J. , & Cormican, L. (2016). Serum vitamin D is significantly inversely associated with disease severity in caucasian adults with obstructive sleep apnea syndrome. Sleep, 39 (2), 293–300. 10.5665/sleep.5430 26414899 PMC4712400

[fsn31867-bib-0026] Khoo, A. L. , Chai, L. Y. , Koenen, H. J. , Sweep, F. C. , Joosten, I. , Netea, M. G. , & van der Ven, A. J. (2011). Regulation of cytokine responses by seasonality of vitamin D status in healthy individuals. Clinical and Experimental Immunology, 164 (1), 72–79. 10.1111/j.1365-2249.2010.04315.x 21323660 PMC3074219

[fsn31867-bib-0027] Kochran, G. ( 1954). The combination of estimates from different experiments. Biometrics, 10 (1), 101 10.2307/3001666

[fsn31867-bib-0028] Kulie, T. , Groff, A. , Redmer, J. , Hounshell, J. , & Schrager, S. (2010). Vitamin D: An evidence‐based review. Journal of the American Board of Family Medicine, 22 (6), 698–706. 10.3122/jabfm.2010.01.090256 19897699

[fsn31867-bib-0029] Lau, E. M. C. , Sambrook, P. , Seeman, E. , Leong, K. H. , Leung, P. C. , & Delmas, P. (2006). Guidelines for diagnosing, prevention and treatment of osteoporosis in Asia. APLAR Journal of Rheumatology, 9 (1), 24–36. 10.1111/j.1479-8077.2006.00161.x

[fsn31867-bib-0030] Lee, P. , Greenfield, J. R. , & Campbell, L. V. (2009). Vitamin D insufficiency–a novel mechanism of statin‐induced myalgia? Clinical Endocrinology ‐ Oxford, 71 (1), 154–155. 10.1111/j.1365-2265.2008.03448.x 19178510

[fsn31867-bib-0031] Liguori, C. , Romigi, A. , Izzi, F. , Mercuri, N. B. , Cordella, A. , Tarquini, E. , … Placidi, F. (2015). Continuous positive airway pressure treatment increases serum vitamin D levels in male patients with obstructive sleep apnea. Journal of Clinical Sleep Medicine, 11 (6), 603–607. 10.5664/jcsm.4766 25766695 PMC4442220

[fsn31867-bib-0032] Majid, M. S. , Ahmad, H. S. , Bizhan, H. , Hosein, H. Z. M. , & Mohammad, A. (2018). The effect of vitamin D supplement on the score and quality of sleep in 20–50 year‐old people with sleep disorders compared with control group. Nutritional Neuroscience, 21 (7), 511–519. 10.1080/1028415x.2017.1317395 28475473

[fsn31867-bib-0033] Marwaha, R. K. , & Dabas, A. (2019). Interventions for prevention and control of epidemic of vitamin D deficiency. Indian Journal of Pediatrics, 86 (6), 532–537. 10.1007/s12098-019-02857-z 30648226

[fsn31867-bib-0034] Mason, C. , de Dieu Tapsoba, J. , Duggan, C. , Wang, C. Y. , Korde, L. , & McTiernan, A. (2016). Repletion of vitamin D associated with deterioration of sleep quality among postmenopausal women. Preventive Medicine, 93, 166–170. 10.1016/j.ypmed.2016.09.035 27687537 PMC5118122

[fsn31867-bib-0035] McCarty, D. E. , Chesson, A. L. Jr , Jain, S. K. , & Marino, A. A. (2014). The link between vitamin D metabolism and sleep medicine. Sleep Medicine Reviews, 18 (4), 311–319. 10.1016/j.smrv.2013.07.001 24075129

[fsn31867-bib-0036] McDonagh, M. S. , Holmes, R. , & Hsu, F. (2019). Pharmacologic treatments for sleep disorders in children: A systematic review. Journal of Child Neurology, 34 (5), 237–247. 10.1177/0883073818821030 30674203

[fsn31867-bib-0037] Mete, T. , Yalcin, Y. , Berker, D. , Ciftci, B. , Guven, S. F. , Topaloglu, O. , … Guler, S. (2013). Obstructive sleep apnea syndrome and its association with vitamin D deficiency. Journal of Endocrinological Investigation, 36 (9), 681–685. 10.3275/8923 23558409

[fsn31867-bib-0038] Mithal, A. , Bansal, B. , Kyer, C. S. , & Ebeling, P. (2014). The Asia‐Pacific Regional Audit‐Epidemiology, Costs, and Burden of Osteoporosis in India 2013: A report of International Osteoporosis Foundation. Indian Journal of Endocrinology and Metabolism, 18 (4), 449–454. 10.4103/2230-8210.137485 25143898 PMC4138897

[fsn31867-bib-0039] Neves, P. D. , Graciolli, F. G. , Oliveira, I. B. , Bridi, R. A. , Moyses, R. M. , & Elias, R. M. (2017). Effect of mineral and bone metabolism on restless legs syndrome in hemodialysis patients. Journal of Clinical Sleep Medicine, 13 (1), 89–94. 10.5664/jcsm.6396 28173916 PMC5181620

[fsn31867-bib-0040] Pazarli, A. C. , Ekiz, T. , & Inonu Koseoglu, H. (2019). Association between 25‐hydroxyvitamin D and bone mineral density in people with obstructive sleep apnea syndrome. Journal of Clinical Densitometry, 22 (1), 39–46. 10.1016/j.jocd.2018.10.001 30396726

[fsn31867-bib-0041] Prietl, B. , Treiber, G. , Pieber, T. R. , & Amrein, K. (2013). Vitamin D and immune function. Nutrients, 5 (7), 2502–2521. Retrieved from . . . . http://europepmc.org/abstract/MED/23857223http://europepmc.org/articles/PMC3738984?pdf=renderhttp://europepmc.org/articles/PMC3738984https://www.ncbi.nlm.nih.gov/pmc/articles/pmid/23857223/?tool=EBIhttps://www.ncbi.nlm.nih.gov/pmc/articles/pmid/23857223/pdf/?tool=EBI. 10.3390/nu5072502 23857223 PMC3738984

[fsn31867-bib-0042] Qiao, Y. , Wang, B. , Yang, J.‐J. , Fan, Y.‐F. , Guo, Q. , Dou, Z.‐J. , … Gao, X.‐L. (2018). Bone metabolic markers in patients with obstructive sleep apnea syndrome. Chinese Medical Journal, 131 (16), 1898–1903. 10.4103/0366-6999.238149 30082519 PMC6085856

[fsn31867-bib-0043] Reid, D. , Morton, R. , Salkeld, L. , & Bartley, J. (2011). Vitamin D and tonsil disease–preliminary observations. International Journal of Pediatric Otorhinolaryngology, 75 (2), 261–264. 10.1016/j.ijporl.2010.11.012 21131064

[fsn31867-bib-0044] Riemann, D. (2009). Does effective management of sleep disorders reduce depressive symptoms and the risk of depression? Drugs, 69 (Suppl 2), 43–64. 10.2165/11531130-000000000-00000 20047350

[fsn31867-bib-0045] Saper, C. B. , Scammell, T. E. , & Lu, J. (2005). Hypothalamic regulation of sleep and circadian rhythms. Nature, 437 (7063), 1257–1263. 10.1038/nature04284 16251950

[fsn31867-bib-0046] Shiue, I. (2013). Low vitamin D levels in adults with longer time to fall asleep: US NHANES, 2005–2006. International Journal of Cardiology, 168 (5), 5074–5075. 10.1016/j.ijcard.2013.07.195 23938219

[fsn31867-bib-0047] Terzi, R. , & Yilmaz, Z. (2016). Bone mineral density and changes in bone metabolism in patients with obstructive sleep apnea syndrome. Journal of Bone and Mineral Metabolism, 34 (4), 475–481. 10.1007/s00774-015-0691-1 26204846

[fsn31867-bib-0048] Toujani, S. , Kaabachi, W. , Mjid, M. , Hamzaoui, K. , Cherif, J. , & Beji, M. (2017). Vitamin D deficiency and interleukin‐17 relationship in severe obstructive sleep apnea‐hypopnea syndrome. Annals of Thoracic Medicine, 12 (2), 107–113. 10.4103/atm.ATM_301_16 28469721 PMC5399684

[fsn31867-bib-0049] Tsou, M.‐T. (2013). Prevalence and risk factors for insomnia in community‐dwelling elderly in northern Taiwan. Journal of Clinical Gerontology and Geriatrics, 4 (3), 75–79. 10.1016/j.jcgg.2013.02.002

[fsn31867-bib-0050] Tufik, S. , Andersen, M. L. , Bittencourt, L. R. , & Mello, M. T. (2009). Paradoxical sleep deprivation: Neurochemical, hormonal and behavioral alterations. Evidence from 30 years of research. Anais da Academia Brasileira de Ciências, 81 (3), 521–538. 10.1590/S0001-37652009000300016 19722021

[fsn31867-bib-0051] Uygur, F. , Baki, A. E. , Tanriverdi, H. , Ornek, T. , & Atalay, F. (2016). Serum vitamin D and parathyroid hormone levels in patients with obstructive sleep apnea syndrome. Istanbul Medical Journal, 17 (2), 64–67. 10.5152/imj.2016.55265

[fsn31867-bib-0052] Wali, S. , Alsafadi, S. , Abaalkhail, B. , Ramadan, I. , Abulhamail, B. , Kousa, M. , … Hamed, M. (2018). The association between vitamin D level and restless legs syndrome: A population‐based case‐control study. Journal of Clinical Sleep Medicine, 14 (4), 557–564. 10.5664/jcsm.7044 29609719 PMC5886433

[fsn31867-bib-0053] Xie, Z. , Chen, F. , Li, W. A. , Geng, X. , Li, C. , Meng, X. , … Yu, F. (2017). A review of sleep disorders and melatonin. Neurological Research, 39 (6), 559–565. 10.1080/01616412.2017.1315864 28460563

[fsn31867-bib-0054] Zhao, K. , Luan, X. , Liu, Y. , Tu, X. , Chen, H. , Shen, H. , … He, J. (2017). Low serum 25‐hydroxyvitamin D concentrations in chronic insomnia patients and the association with poor treatment outcome at 2 months. Clinica Chimica Acta, 475, 147–151. 10.1016/j.cca.2017.10.024 29080688

[fsn31867-bib-0055] Zicari, A. M. , Occasi, F. , Di Mauro, F. , Lollobrigida, V. , Di Fraia, M. , Savastano, V. , … Duse, M. (2016). Mean platelet volume, vitamin D and C reactive protein levels in normal weight children with primary snoring and obstructive sleep apnea syndrome. PLoS One, 11 (4), e0152497 10.1371/journal.pone.0152497 27054959 PMC4824489

