# Supplementary figures and images for: A meta‐analysis: Does vitamin D play a promising role in sleep disorders?
Source: Food Sci Nutr. 2020 Sep 9;8(10):5696–709. doi: 10.1002/fsn3.1867 (PMC7590291; doi:10.1002/fsn3.1867)

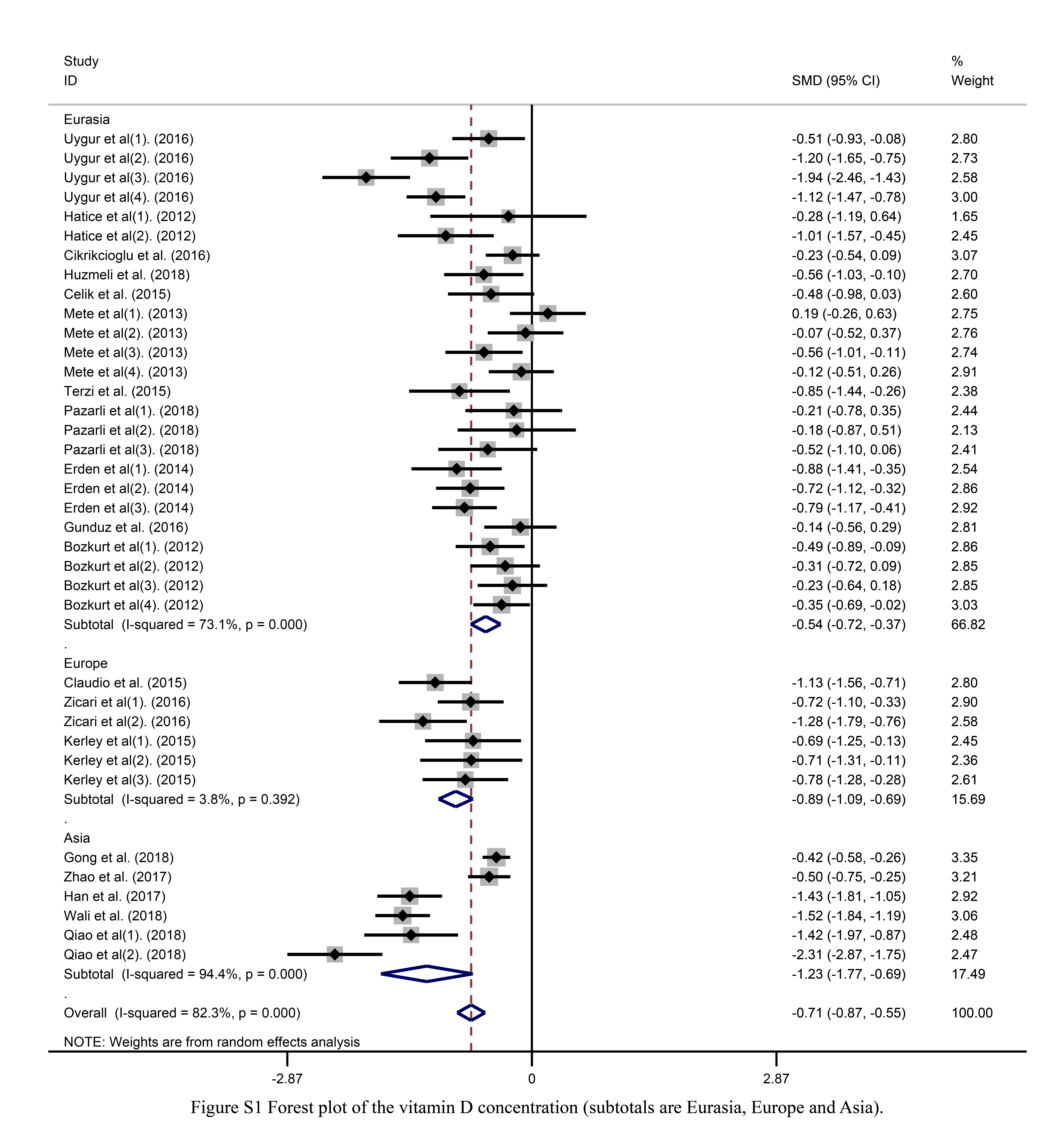

Supplement: Supplementary file 1 — FigS1 [file FSN3-8-5696-s001.tif]

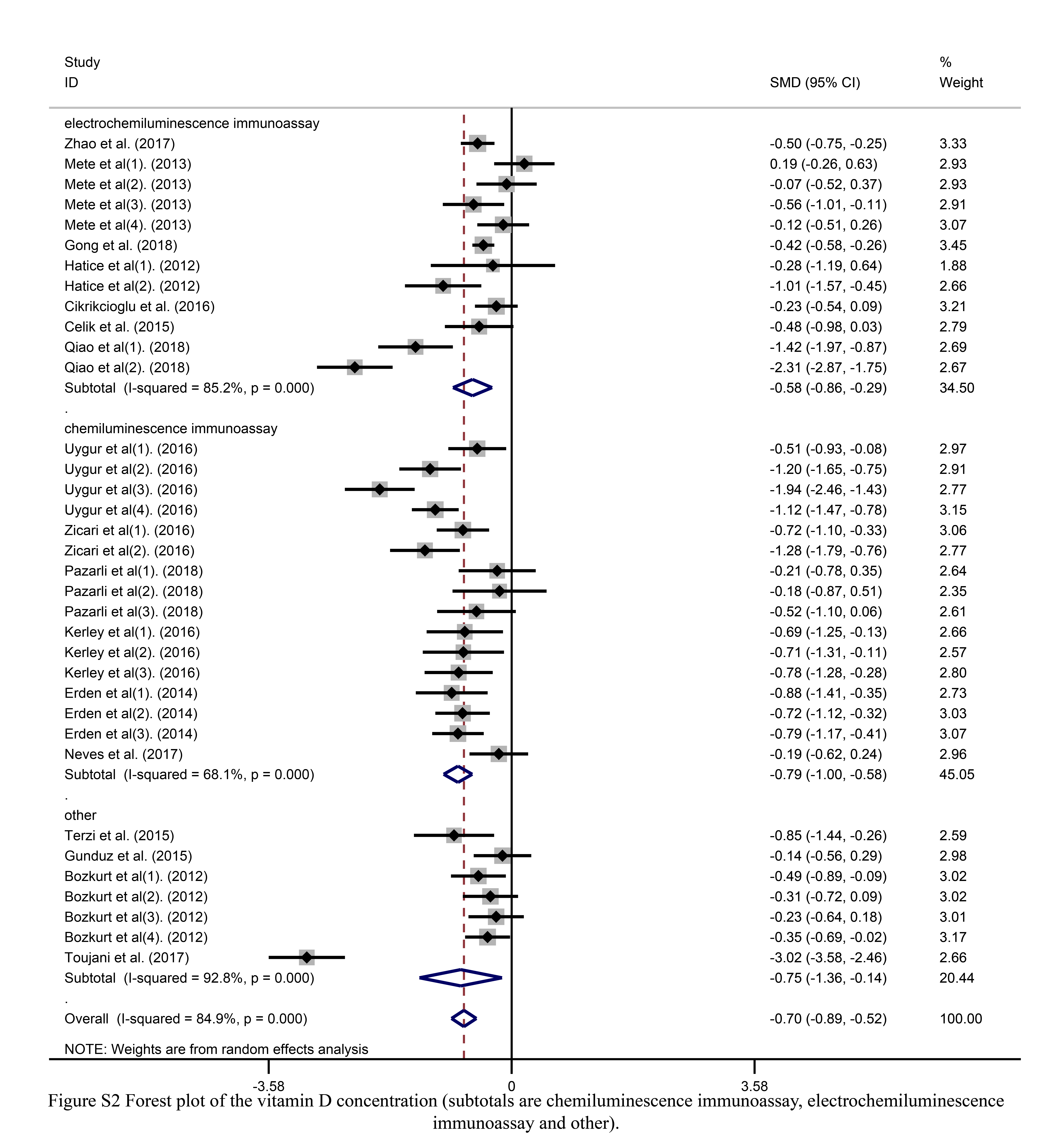

Supplement: Supplementary file 2 — FigS2 [file FSN3-8-5696-s002.tif]

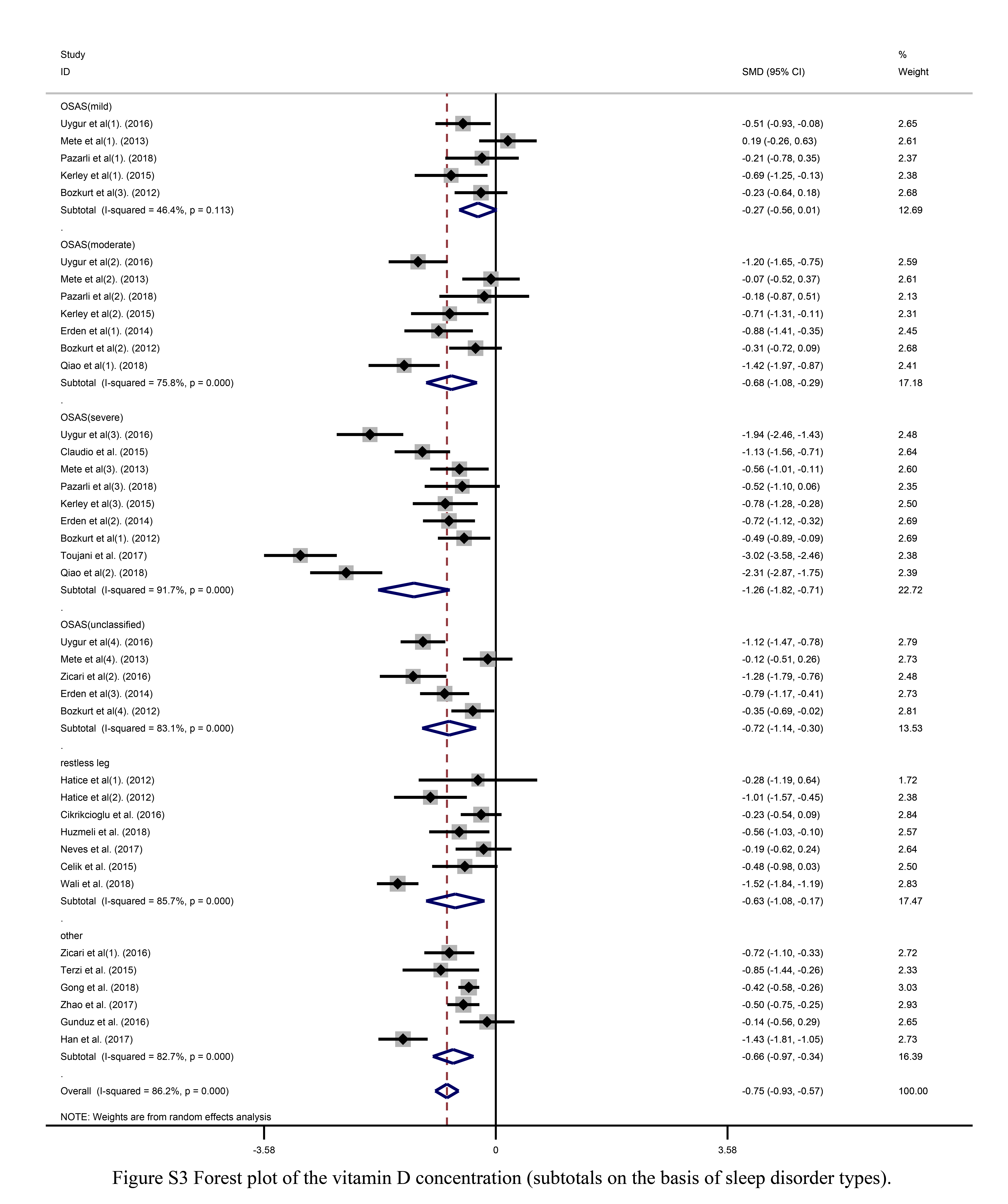

Supplement: Supplementary file 3 — FigS3 [file FSN3-8-5696-s003.tif]

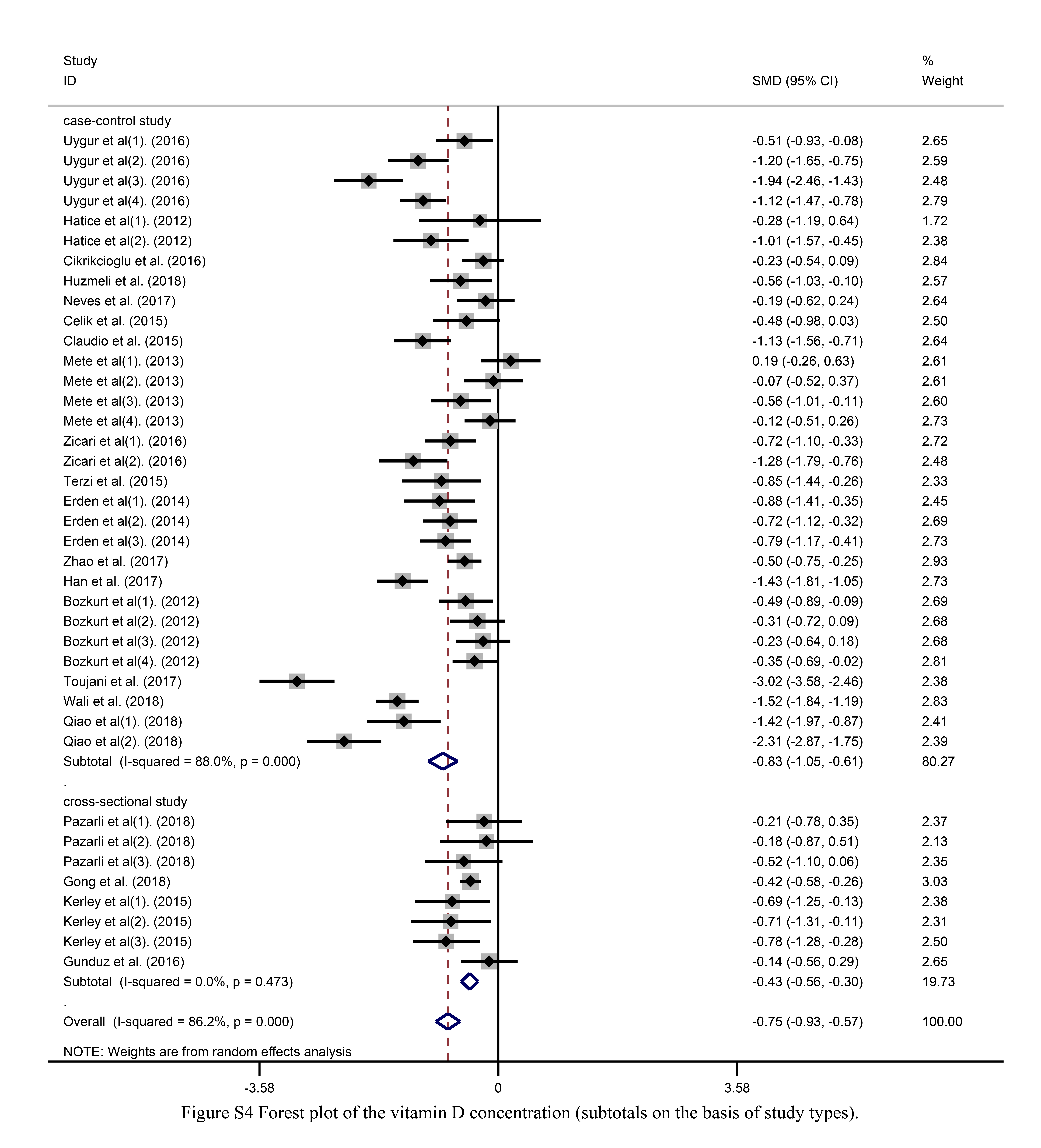

Supplement: Supplementary file 4 — FigS4 [file FSN3-8-5696-s004.tif]

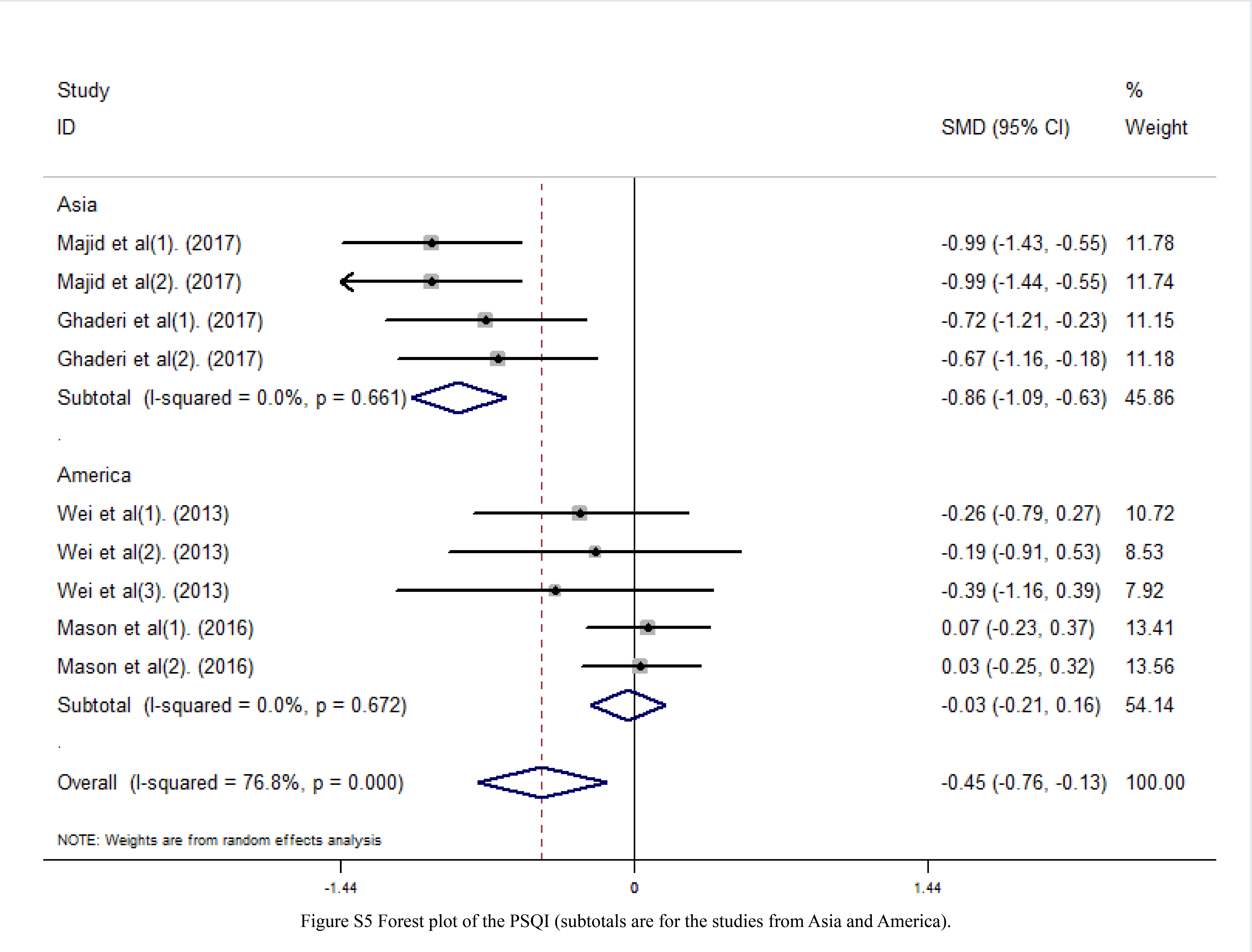

Supplement: Supplementary file 5 — FigS5 [file FSN3-8-5696-s005.tif]
