# Supplementary material for: A meta‐analysis: Does vitamin D play a promising role in sleep disorders?
Source: Food Sci Nutr. 2020 Sep 9;8(10):5696–709. doi: 10.1002/fsn3.1867 (PMC7590291; doi:10.1002/fsn3.1867)
Supplement: Supplementary file 8 — TableS1 [file FSN3-8-5696-s008.docx]

**supplementation materials**

**Kerley et al. 2015**

| Study type | Cross-sectional study | | |
| --- | --- | --- | --- |
| Participants | Adults with obstructive sleep apnea syndrome(OSAS)  N:75 cases, 31 controls  Mean age in years (SD): 54.00(19.00) case(mild), 57.00(17.00) case(moderate), 55.50 (17.00) case (severe), 53.00(19.00) control.  Gender: 17 males/5 females case(mild), 12 males/6 females case(moderate), 28 males/7 females case(severe), 16 males /15 females control.  Location: Ireland | | |
| Comparison | Comparison: patients with sleep disorder vs. control  Case: Adults with OSAS (mild, moderate and severe)  Control: non-OSAS. | | |
| Outcomes | Main study outcome: vitamin D levels of adults with OSAS and non-OSAS, Vitamin D levels and relationships to demographics.  Available outcomes: vitamin D levels of adults with OSAS(different degree) and non-OSAS. | | |
| ***Risk of bias*** | | | |
| **Bias** | | **Authors’ judgment** | **Support for judgment** |
| Is the case definition adequate(**Selection**) | | 1 | yes, with independent validation |
| Representativeness of the cases(**Selection**) | | 1 | consecutive or obviously representative series of cases |
| Selection of Controls(**Selection**) | | 0 | hospital controls |
| Definition of Controls(**Selection**) | | 1 | no history of disease (endpoint) |
| Comparability of cases and controls on the basis of the design or analysis(**Comparability**) | | 2 | study controls for age, gender and other factors. |
| Ascertainment of exposure(**Exposure**) | | 1 | secure record |
| Same method of ascertainment for cases and controls(**Exposure**) | | 1 | yes |
| Non-Response rate(**Exposure**) | | 1 | same rate for both groups |

**Hatice et al. 2012**

| Study type | Case-control study | | |
| --- | --- | --- | --- |
| Participants | Patients with restless legs syndrome(RLS)  N: 36 cases, 38 controls  Mean age in years (SD): 41.50(6.27) case(male), 39.64(7.65) case(female), 36.45(8.43) control (male), 37.96(8.33) control (female).  Gender: 8 males/28 females case, 11 males/27 females control.  Location: Turkey | | |
| Comparison | Comparison: patients with sleep disorder vs. control  Case: Patients with RLS  Control: non-RLS | | |
| Outcomes | Main study outcome: Comparison of biochemical variables between RLS patients and healthy controls, correlation between concentration of vitamin D and IRLS score in female RLS patients.  Available outcomes: vitamin D levels of patients with RLS and control. | | |
| ***Risk of bias*** | | | |
| **Bias** | | **Authors’ judgment** | **Support for judgment** |
| Is the case definition adequate(**Selection**) | | 1 | yes, with independent validation |
| Representativeness of the cases(**Selection**) | | 1 | consecutive or obviously representative series of cases |
| Selection of Controls(**Selection**) | | 0 | hospital controls |
| Definition of Controls(**Selection**) | | 1 | no history of disease (endpoint) |
| Comparability of cases and controls on the basis of the design or analysis(**Comparability**) | | 2 | study controls for age, gender and other factors. |
| Ascertainment of exposure(**Exposure**) | | 1 | secure record |
| Same method of ascertainment for cases and controls(**Exposure**) | | 1 | yes |
| Non-Response rate(**Exposure**) | | 1 | same rate for both groups |

**Cikrikcioglu et al. 2016**

| Study type | Case-control study | | |
| --- | --- | --- | --- |
| Participants | Patients with restless legs syndrome(RLS)  N: 78 cases, 78 controls  Mean age in years (SD): 46.45(11.26) case, 45.01(12.27) control.  Gender: 0 males/78 females case, 0 males/78 females control.  Location: Turkey | | |
| Comparison | Comparison: patients with sleep disorder vs. control  Case: Patients with RLS  Control: non-RLS | | |
| Outcomes | Main study outcome: Demographic features, medicine regularly used, RLS severity, vitamin D, t score status, rutin blood analysis results, bone biomarkers, and bone mineral density findings among the participants of the study.  Available outcomes: vitamin D levels of patients with RLS and control. | | |
| ***Risk of bias*** | | | |
| **Bias** | | **Authors’ judgment** | **Support for judgment** |
| Is the case definition adequate(**Selection**) | | 1 | yes, with independent validation |
| Representativeness of the cases(**Selection**) | | 1 | consecutive or obviously representative series of cases |
| Selection of Controls(**Selection**) | | 0 | no description |
| Definition of Controls(**Selection**) | | 1 | no history of disease (endpoint) |
| Comparability of cases and controls on the basis of the design or analysis(**Comparability**) | | 2 | study controls for age, BMI and other factors. |
| Ascertainment of exposure(**Exposure**) | | 1 | secure record |
| Same method of ascertainment for cases and controls(**Exposure**) | | 1 | yes |
| Non-Response rate(**Exposure**) | | 1 | same rate for both groups |

**Huzmeli et al. 2018**

| Study type | Case-control study | | |
| --- | --- | --- | --- |
| Participants | Patients with restless legs syndrome(RLS)  N: 33 cases, 42 controls  Mean age in years (SD): 59.60(12.90) case, 56.65(15.77) control.  Gender: 9 males/24 females case, 26 males/16 females control.  Location: Turkey | | |
| Comparison | Comparison: patients with sleep disorder vs. control  Case: Patients with RLS  Control: non-RLS | | |
| Outcomes | Main study outcome: Laboratory data and features in patients with RLS and non-RLS.  Available outcomes: vitamin D levels of patients with RLS and control. | | |
| ***Risk of bias*** | | | |
| **Bias** | | **Authors’ judgment** | **Support for judgment** |
| Is the case definition adequate(**Selection**) | | 1 | yes, with independent validation |
| Representativeness of the cases(**Selection**) | | 1 | consecutive or obviously representative series of cases |
| Selection of Controls(**Selection**) | | 0 | no description |
| Definition of Controls(**Selection**) | | 1 | no history of disease (endpoint) |
| Comparability of cases and controls on the basis of the design or analysis(**Comparability**) | | 1 | study only controls age between case and control. |
| Ascertainment of exposure(**Exposure**) | | 1 | secure record |
| Same method of ascertainment for cases and controls(**Exposure**) | | 1 | yes |
| Non-Response rate(**Exposure**) | | 1 | same rate for both groups |

**Neves et al. 2017**

| Study type | Case-control study | | |
| --- | --- | --- | --- |
| Participants | Patients with restless legs syndrome(RLS)  N: 29 cases, 72 controls  Mean age in years (SD): 47.00(18.00) case, 45.00 (15.00) control.  Gender: 11 males/18 females case, 43 males/29 females control.  Location: Brazil | | |
| Comparison | Comparison: patients with sleep disorder vs. control  Case: Presence of RLS  Control: Absence of RLS | | |
| Outcomes | Main study outcome: Characteristics of patients according to restless legs syndrome presence or absence, relationship between scores of RLS severity and markers of mineral and bone metabolism, differences in the percentage of hyperphosphatemia, levels of fibroblast growth factor-23 (FGF-23), and 25(OH)-vitamin D defciency when comparing patients with severe/very severe and those with mild/moderate RLS symptoms.  Available outcomes: vitamin D levels of patients with RLS and control. | | |
| ***Risk of bias*** | | | |
| **Bias** | | **Authors’ judgment** | **Support for judgment** |
| Is the case definition adequate(**Selection**) | | 1 | yes, with independent validation |
| Representativeness of the cases(**Selection**) | | 1 | consecutive or obviously representative series of cases |
| Selection of Controls(**Selection**) | | 0 | hospital controls |
| Definition of Controls(**Selection**) | | 1 | no history of disease (endpoint) |
| Comparability of cases and controls on the basis of the design or analysis(**Comparability**) | | 2 | study controls for age, gender and other factors. |
| Ascertainment of exposure(**Exposure**) | | 1 | secure record |
| Same method of ascertainment for cases and controls(**Exposure**) | | 1 | yes |
| Non-Response rate(**Exposure**) | | 1 | same rate for both groups |

**Celik et al. 2015**

| Study type | Case-control study | | |
| --- | --- | --- | --- |
| Participants | Patients with restless legs syndrome(RLS)  N: 31 cases, 31 controls  Mean age in years (SD): 43.61(10.51) case, 45.64(14.43) control.  Gender: 0 males/31 females case, 0 males/31 females control.  Location: Turkey | | |
| Comparison | Comparison: patients with sleep disorder vs. control  Case: Patients with RLS  Control: non-RLS | | |
| Outcomes | Main study outcome: Menopause, comorbidities, smoking, and working status of subjects included in the study, blood analysis in both groups.  Available outcomes: vitamin D levels of patients with RLS and control. | | |
| ***Risk of bias*** | | | |
| **Bias** | | **Authors’ judgment** | **Support for judgment** |
| Is the case definition adequate(**Selection**) | | 1 | yes, with independent validation |
| Representativeness of the cases(**Selection**) | | 1 | consecutive or obviously representative series of cases |
| Selection of Controls(**Selection**) | | 0 | hospital controls |
| Definition of Controls(**Selection**) | | 1 | no history of disease (endpoint) |
| Comparability of cases and controls on the basis of the design or analysis(**Comparability**) | | 2 | study controls for age, BMI and other factors. |
| Ascertainment of exposure(**Exposure**) | | 1 | secure record |
| Same method of ascertainment for cases and controls(**Exposure**) | | 1 | yes |
| Non-Response rate(**Exposure**) | | 1 | same rate for both groups |

**Wali et al. 2018**

| Study type | Case-control study | | |
| --- | --- | --- | --- |
| Participants | Patients with restless legs syndrome(RLS)  N: 78 cases, 123 controls  Mean age in years (SD): 43.79 (6.04) case, 44.75 (9.59) control.  Gender: 38 males/40 females case, 59 males/64 females control.  Location: Saudi Arabia | | |
| Comparison | Comparison: patients with sleep disorder vs. control  Case: Patients with RLS  Control: non-RLS | | |
| Outcomes | Main study outcome: Biochemical characteristics of the study participants.  Available outcomes: vitamin D levels of patients with RLS and control. | | |
| ***Risk of bias*** | | | |
| **Bias** | | **Authors’ judgment** | **Support for judgment** |
| Is the case definition adequate(**Selection**) | | 1 | yes, with independent validation |
| Representativeness of the cases(**Selection**) | | 1 | consecutive or obviously representative series of cases |
| Selection of Controls(**Selection**) | | 0 | hospital controls |
| Definition of Controls(**Selection**) | | 1 | no history of disease (endpoint) |
| Comparability of cases and controls on the basis of the design or analysis(**Comparability**) | | 2 | study controls for age, BMI and other factors. |
| Ascertainment of exposure(**Exposure**) | | 1 | secure record |
| Same method of ascertainment for cases and controls(**Exposure**) | | 1 | yes |
| Non-Response rate(**Exposure**) | | 1 | same rate for both groups |

**Claudio et al. 2015**

| Study type | Case-control study | | |
| --- | --- | --- | --- |
| Participants | Patients with severe OSA  N:90 cases, 32 controls  Mean age in years (SD): 61.10(12.68) case, 59.12 (8.02) control.  Gender: 60 males/24 females case, 22 males /10 females control.  Location: Italy | | |
| Comparison | Comparison: patients with sleep disorder vs. control  Case: Patients with severe OSA  Control: a population of volunteers with low sun exposure (indoor workers) | | |
| Outcomes | Main study outcome: Vitamin D, PTH and calcium levels in OSA patient group and controls, correlations between clinical, polygraphic, and serum data in patients with OSA, vitamin D, PTH, and calcium levels in patient groups after nCPAP therapy.  Available outcomes: vitamin D levels of subjects with severe OSA and control. | | |
| ***Risk of bias*** | | | |
| **Bias** | | **Authors’ judgment** | **Support for judgment** |
| Is the case definition adequate(**Selection**) | | 1 | yes, with independent validation |
| Representativeness of the cases(**Selection**) | | 1 | consecutive or obviously representative series of cases |
| Selection of Controls(**Selection**) | | 1 | community controls |
| Definition of Controls(**Selection**) | | 1 | no history of disease (endpoint) |
| Comparability of cases and controls on the basis of the design or analysis(**Comparability**) | | 2 | study controls for age, gender and other factors. |
| Ascertainment of exposure(**Exposure**) | | 1 | secure record |
| Same method of ascertainment for cases and controls(**Exposure**) | | 1 | yes |
| Non-Response rate(**Exposure**) | | 1 | same rate for both groups |

**Toujani et al. 2017**

| Study type | Case-control study | | |
| --- | --- | --- | --- |
| Participants | Patients with severe OSA  N:92 cases, 30 controls  Mean age in years (SD): 52.30 (12.70) case, 45.70 (14.70) control.  Gender: 48 males/44 females case, 17 males /13 females control.  Location: Tunisia | | |
| Comparison | Comparison: patients with sleep disorder vs. control  Case: Patients with severe OSA  Control: The controls were volunteers who had no sleep disorders and similar to the patients group in terms of age, sex, and body mass index (BMI). | | |
| Outcomes | Main study outcome: interleukin-17 and Vitamin D serum levels  Available outcomes: vitamin D levels of subjects with severe OSA and control. | | |
| ***Risk of bias*** | | | |
| **Bias** | | **Authors’ judgment** | **Support for judgment** |
| Is the case definition adequate(**Selection**) | | 1 | yes, with independent validation |
| Representativeness of the cases(**Selection**) | | 1 | consecutive or obviously representative series of cases |
| Selection of Controls(**Selection**) | | 1 | community controls |
| Definition of Controls(**Selection**) | | 1 | no history of disease (endpoint) |
| Comparability of cases and controls on the basis of the design or analysis(**Comparability**) | | 2 | study controls for age, gender and other factors. |
| Ascertainment of exposure(**Exposure**) | | 1 | secure record |
| Same method of ascertainment for cases and controls(**Exposure**) | | 1 | yes |
| Non-Response rate(**Exposure**) | | 1 | same rate for both groups |

**Mete et al. 2013**

| Study type | Case-control study | | |
| --- | --- | --- | --- |
| Participants | Patients with OSAS  N:150cases, 32 controls  Mean age in years (SD): 46.58(9.37) case(mild), 47.64(7.22) case(moderate), 47.40(9.48) case (severe), 46.94(8.10) control.  Gender: 25 males/25 females case(mild), 25 males/25 females case(moderate), 25 males/25 females case(severe), 16 males /16 females control.  Location: Turkey | | |
| Comparison | Comparison: patients with sleep disorder vs. control  Case: Patients with OSAS(mild, moderate, severe and unclassified)  Control: non-OSAS | | |
| Outcomes | Main study outcome: Polysomnography findings of patients and control group, evaluation of vitamin D levels of patients and control group.  Available outcomes: vitamin D levels of subjects with OSAS (different degree) and control. | | |
| ***Risk of bias*** | | | |
| **Bias** | | **Authors’ judgment** | **Support for judgment** |
| Is the case definition adequate(**Selection**) | | 1 | yes, with independent validation |
| Representativeness of the cases(**Selection**) | | 1 | consecutive or obviously representative series of cases |
| Selection of Controls(**Selection**) | | 0 | hospital controls |
| Definition of Controls(**Selection**) | | 1 | no history of disease (endpoint) |
| Comparability of cases and controls on the basis of the design or analysis(**Comparability**) | | 2 | study controls for age, gender and other factors. |
| Ascertainment of exposure(**Exposure**) | | 1 | secure record |
| Same method of ascertainment for cases and controls(**Exposure**) | | 1 | yes |
| Non-Response rate(**Exposure**) | | 1 | same rate for both groups |

**Zicari et al. 2016**

| Study type | Case-control study | | |
| --- | --- | --- | --- |
| Participants | Patients with OSAS and Primary Snoring(PS)  N: 67 cases, 70 controls  Mean age in years (SD): 9.00(1.75) case(PS), 7.62(3.09) case(OSAS), 9.04(3.91) control.  Gender: 29 males/16 females case(PS), 15 males/7 females case(OSAS), 40 males /30 females control.  Location: Italy | | |
| Comparison | Comparison: patients with sleep disorder vs. control  Case: Patients with OSAS and PS  Control: healthy controls | | |
| Outcomes | Main study outcome: Comparison of the Platelet number, Mean Platelet Volume(MPV), C Reactive Protein(CRP) and vitamin D of patients with OSAS, PS and control.  Available outcomes: vitamin D levels of patients with OSAS, PS and control. | | |
| ***Risk of bias*** | | | |
| **Bias** | | **Authors’ judgment** | **Support for judgment** |
| Is the case definition adequate(**Selection**) | | 1 | yes, with independent validation |
| Representativeness of the cases(**Selection**) | | 1 | consecutive or obviously representative series of cases |
| Selection of Controls(**Selection**) | | 0 | hospital controls |
| Definition of Controls(**Selection**) | | 1 | no history of disease (endpoint) |
| Comparability of cases and controls on the basis of the design or analysis(**Comparability**) | | 2 | study controls for age, gender and other factors. |
| Ascertainment of exposure(**Exposure**) | | 1 | secure record |
| Same method of ascertainment for cases and controls(**Exposure**) | | 1 | yes |
| Non-Response rate(**Exposure**) | | 1 | same rate for both groups |

**Terzi et al. 2015**

| Study type | Case-control study | | |
| --- | --- | --- | --- |
| Participants | Patients with OSAS  N:30 cases, 20 controls  Mean age in years (SD): 52.37(8.58) case, 50.60(10.84) control.  Gender: 30 males/0 females case, 20 males /0 females control.  Location: Turkey | | |
| Comparison | Comparison: patients with sleep disorder vs. control  Case: Patients with OSAS  Control: non-OSAS. | | |
| Outcomes | Main study outcome: Evaluation of *T*-scores, bone mineral density, and the laboratory parameters of patients with OSAS and non-OSAS, the association between AHI values, mean O_2_, minimum O_2_, bone turnover markers, and bone mineral density.  Available outcomes: vitamin D levels of patients with OSAS and non-OSAS. | | |
| ***Risk of bias*** | | | |
| **Bias** | | **Authors’ judgment** | **Support for judgment** |
| Is the case definition adequate(**Selection**) | | 1 | yes, with independent validation |
| Representativeness of the cases(**Selection**) | | 1 | consecutive or obviously representative series of cases |
| Selection of Controls(**Selection**) | | 0 | hospital controls |
| Definition of Controls(**Selection**) | | 1 | no history of disease (endpoint) |
| Comparability of cases and controls on the basis of the design or analysis(**Comparability**) | | 1 | study only controls age between case and control. |
| Ascertainment of exposure(**Exposure**) | | 1 | secure record |
| Same method of ascertainment for cases and controls(**Exposure**) | | 1 | yes |
| Non-Response rate(**Exposure**) | | 1 | same rate for both groups |

**Pazarli et al. 2018**

| Study type | Cross-sectional study | | |
| --- | --- | --- | --- |
| Participants | Patients with OSAS  N: 68 cases, 21 controls  Mean age in years (SD): 46.50(11.50) case(mild), 51.00(13.80) case(moderate), 50.40(11.90) case (severe), 42.10(1.80) control.  Gender: 17 males/11 females case(mild), 5 males/8 females case(moderate), 24 males/3 females case(severe), 10 males /11 females control.  Location: Turkey | | |
| Comparison | Comparison: patients with sleep disorder vs. control  Case: Patients with OSAS(mild, moderate and severe)  Control: normal patients | | |
| Outcomes | Main study outcome: Comparison of the demographic, clinical features and laboratory parameters of patients with OSAS (different degree) and control.  Available outcomes: vitamin D levels of patients with OSAS (different degree) and control. | | |
| ***Risk of bias*** | | | |
| **Bias** | | **Authors’ judgment** | **Support for judgment** |
| Is the case definition adequate(**Selection**) | | 1 | yes, with independent validation |
| Representativeness of the cases(**Selection**) | | 1 | consecutive or obviously representative series of cases |
| Selection of Controls(**Selection**) | | 0 | hospital controls |
| Definition of Controls(**Selection**) | | 1 | no history of disease (endpoint) |
| Comparability of cases and controls on the basis of the design or analysis(**Comparability**) | | 1 | study only controls age between case and control. |
| Ascertainment of exposure(**Exposure**) | | 1 | secure record |
| Same method of ascertainment for cases and controls(**Exposure**) | | 1 | yes |
| Non-Response rate(**Exposure**) | | 1 | same rate for both groups |

**Gong et al. 2018**

| Study type | Cross-sectional study | | |
| --- | --- | --- | --- |
| Participants | Adolescents aged 8–14 years in apparently good health  N:262 cases, 353 controls  Mean age in years (SD): 12.22 (1.75) case, 10.24 (1.73) control.  Gender: 128 males/134 females case, 205 males /148 females control.  Location: China | | |
| Comparison | Comparison: patients with sleep disorder vs. control  Case: Adolescents aged 8–14 years(<9.0 h/day)  Control: Adolescents aged 8–14 years(9.0–9.9 h/day) | | |
| Outcomes | Main study outcome: Characteristics of the study subjects by sleep duration, correlation between sleep duration and 25(OH)D level in 800 subjects, odds ratio (OR) and 95% confidence interval (CI) for insufficient sleep (<9 h/day) by categories of 25(OH)D levels.  Available outcomes: vitamin D levels of Adolescents(<9.0 h/day) and control(9.0–9.9 h/day). | | |
| ***Risk of bias*** | | | |
| **Bias** | | **Authors’ judgment** | **Support for judgment** |
| Is the case definition adequate(**Selection**) | | 1 | yes, with independent validation |
| Representativeness of the cases(**Selection**) | | 1 | consecutive or obviously representative series of cases |
| Selection of Controls(**Selection**) | | 1 | community controls |
| Definition of Controls(**Selection**) | | 1 | no history of disease (endpoint) |
| Comparability of cases and controls on the basis of the design or analysis(**Comparability**) | | 1 | study only controls gender between case and control. |
| Ascertainment of exposure(**Exposure**) | | 0 | written self report |
| Same method of ascertainment for cases and controls(**Exposure**) | | 1 | yes |
| Non-Response rate(**Exposure**) | | 1 | same rate for both groups |

**Uygur et al. 2016**

| Study type | Case-control study | | |
| --- | --- | --- | --- |
| Participants | Patients with OSAS  N:103 cases, 58 controls  Mean age in years (SD): 46.70(9.20) case, 44.60(9.70) control.  Gender: 54 males/49 females case, 23 males /35 females control.  Location: Turkey | | |
| Comparison | Comparison: patients with sleep disorder vs. control  Case: Patients with OSAS (mild, moderate, severe and unclassified)  Control: non-OSAS. | | |
| Outcomes | Main study outcome: vitamin D and PTH levels of subjects with OSAS and non-OSAS.  Available outcomes: vitamin D levels of subjects with OSAS(different degree) and non-OSAS. | | |
| ***Risk of bias*** | | | |
| **Bias** | | **Authors’ judgment** | **Support for judgment** |
| Is the case definition adequate(**Selection**) | | 1 | yes, with independent validation |
| Representativeness of the cases(**Selection**) | | 1 | consecutive or obviously representative series of cases |
| Selection of Controls(**Selection**) | | 0 | no description |
| Definition of Controls(**Selection**) | | 1 | no history of disease (endpoint) |
| Comparability of cases and controls on the basis of the design or analysis(**Comparability**) | | 2 | study controls for age, gender and other factors. |
| Ascertainment of exposure(**Exposure**) | | 1 | secure record |
| Same method of ascertainment for cases and controls(**Exposure**) | | 1 | yes |
| Non-Response rate(**Exposure**) | | 1 | same rate for both groups |

**Erden et al. 2014**

| Study type | Case-control study | | |
| --- | --- | --- | --- |
| Participants | Patients with OSAS  N:85 cases, 43 controls  Mean age in years (SD): 48.56(9.74) case, 45.00(14.00) control.  Gender: 70 males/15 females case, 21 males /22 females control.  Location: Turkey | | |
| Comparison | Comparison: patients with sleep disorder vs. control  Case: Subjects with OSAS(moderate, severe and unclassified)  Control: healthy volunteers. | | |
| Outcomes | Main study outcome: vitamin D levels of subjects with OSAS and non-OSAS, the independent predictors of OSAS.  Available outcomes: vitamin D levels of subjects with OSAS and non-OSAS. | | |
| ***Risk of bias*** | | | |
| **Bias** | | **Authors’ judgment** | **Support for judgment** |
| Is the case definition adequate(**Selection**) | | 1 | yes, with independent validation |
| Representativeness of the cases(**Selection**) | | 1 | consecutive or obviously representative series of cases |
| Selection of Controls(**Selection**) | | 1 | community controls |
| Definition of Controls(**Selection**) | | 1 | no history of disease (endpoint) |
| Comparability of cases and controls on the basis of the design or analysis(**Comparability**) | | 0 | failure to control any factor between case and control. |
| Ascertainment of exposure(**Exposure**) | | 1 | secure record |
| Same method of ascertainment for cases and controls(**Exposure**) | | 1 | yes |
| Non-Response rate(**Exposure**) | | 1 | same rate for both groups |

**Zhao et al. 2017**

| Study type | Case-control study | | |
| --- | --- | --- | --- |
| Participants | Chronic insomnia patients  N:181 cases, 100 controls  Mean age in years (SD): 43.16(10.78) case, 44.31(10.33) control  Gender: 52 males/129 females case, 32 males /68 females control  Location: China | | |
| Comparison | Comparison: patients with sleep disorder vs. control  Case: chronic insomnia patients.  Control: health control. | | |
| Outcomes | Main study outcome: vitamin D levels of non-response patients, response patients and healthy control.  Dropouts: 40 subjects  Available outcomes: vitamin D levels of chronic insomnia patients and healthy control. | | |
| ***Risk of bias*** | | | |
| **Bias** | | **Authors’ judgment** | **Support for judgment** |
| Is the case definition adequate(**Selection**) | | 1 | yes, with independent validation |
| Representativeness of the cases(**Selection**) | | 1 | consecutive or obviously representative series of cases |
| Selection of Controls(**Selection**) | | 0 | hospital controls |
| Definition of Controls(**Selection**) | | 1 | no history of disease (endpoint) |
| Comparability of cases and controls on the basis of the design or analysis(**Comparability**) | | 2 | study controls for age, gender and other factors. |
| Ascertainment of exposure(**Exposure**) | | 1 | secure record |
| Same method of ascertainment for cases and controls(**Exposure**) | | 1 | yes |
| Non-Response rate(**Exposure**) | | 0 | non respondents described |

**Gunduz et al. 2016**

| Study type | Cross-sectional study | | |
| --- | --- | --- | --- |
| Participants | pregnant women with sleep deprivation  N:58 cases, 34 controls  Mean age in years (SD): 29.70 (4.80) case, 30.50 (4.20) control  Gender: 0 males/58 females case, 0 males /34 females control  Location: Turkey | | |
| Comparison | Comparison: patients with sleep disorder vs. control  Case: pregnant women with poor sleep quality.  Control: pregnant women with good sleep quality. | | |
| Outcomes | Main study outcome: the PSQI of the vitamin D-deficient group and the vitamin D-nondeficient group, the vitamin D levels of the poor sleep quality and the good sleep quality.  Available outcomes: vitamin D levels of pregnant women with poor sleep quality and good sleep quality. | | |
| ***Risk of bias*** | | | |
| **Bias** | | **Authors’ judgment** | **Support for judgment** |
| Is the case definition adequate(**Selection**) | | 1 | yes, with independent validation |
| Representativeness of the cases(**Selection**) | | 1 | consecutive or obviously representative series of cases |
| Selection of Controls(**Selection**) | | 0 | hospital controls |
| Definition of Controls(**Selection**) | | 1 | no history of disease (endpoint) |
| Comparability of cases and controls on the basis of the design or analysis(**Comparability**) | | 2 | study controls for age, gender and other factors. |
| Ascertainment of exposure(**Exposure**) | | 0 | written self report |
| Same method of ascertainment for cases and controls(**Exposure**) | | 1 | yes |
| Non-Response rate(**Exposure**) | | 1 | same rate for both groups |

**Han et al. 2017**

| Study type | Case-control study | | |
| --- | --- | --- | --- |
| Participants | Patients undergoing maintenance hemodialysis  N:88 cases, 53 controls  Mean age in years (SD): 59.70(15.30) case, 62.80(12.50) control  Gender: 52 males/36 females case, 34 males /19 females control  Location: China | | |
| Comparison | Comparison: patients with sleep disorder vs. control  Case: hemodialysis patients with poor sleep quality.  Control: hemodialysis patients with good sleep quality. | | |
| Outcomes | Main study outcome: vitamin D levels of poor sleepers and good sleepers in hemodialysis patients, characteristics associated with sleep disturbance in HD patients.  Available outcomes: vitamin D levels of poor sleepers and good sleepers. | | |
| ***Risk of bias*** | | | |
| **Bias** | | **Authors’ judgment** | **Support for judgment** |
| Is the case definition adequate(**Selection**) | | 1 | yes, with independent validation |
| Representativeness of the cases(**Selection**) | | 1 | consecutive or obviously representative series of cases |
| Selection of Controls(**Selection**) | | 0 | hospital controls |
| Definition of Controls(**Selection**) | | 1 | no history of disease (endpoint) |
| Comparability of cases and controls on the basis of the design or analysis(**Comparability**) | | 2 | study controls for age, gender and other factors. |
| Ascertainment of exposure(**Exposure**) | | 0 | written self report |
| Same method of ascertainment for cases and controls(**Exposure**) | | 1 | yes |
| Non-Response rate(**Exposure**) | | 1 | same rate for both groups |

**Bozkurt et al. 2012**

| Study type | Case-control study | | |
| --- | --- | --- | --- |
| Participants | Subjects with obstructive sleep apnea(OSA)  N:143 cases, 47 controls  Mean age in years (SD): 47.78(10.35) case(mild), 49.79(10.62) case(moderate), 49.66(10.38) case (severe), 42.79(9.55) control.  Gender: 28 males/18 females case(mild), 28 males/19 females case(moderate), 29 males/21 females case(severe), 28 males /19 females control.  Location: Turkey | | |
| Comparison | Comparison: patients with sleep disorder vs. control  Case: Subjects with OSA (mild, moderate, severe and unclassified)  Control: non-OSA. | | |
| Outcomes | Main study outcome: vitamin D levels of subjects with OSA and non-OSA, the correlation of serum vitamin D levels and all other parameters in study subjects after performing the principal component analysis (PCA), comparisons of insulin resistant(IR) patients with non-insulin resistant subjects.  Available outcomes: vitamin D levels of subjects with OSA(different degree) and non-OSA. | | |
| ***Risk of bias*** | | | |
| **Bias** | | **Authors’ judgment** | **Support for judgment** |
| Is the case definition adequate(**Selection**) | | 1 | yes, with independent validation |
| Representativeness of the cases(**Selection**) | | 1 | consecutive or obviously representative series of cases |
| Selection of Controls(**Selection**) | | 0 | no description |
| Definition of Controls(**Selection**) | | 1 | no history of disease (endpoint) |
| Comparability of cases and controls on the basis of the design or analysis(**Comparability**) | | 1 | study only controls gender between case and control. |
| Ascertainment of exposure(**Exposure**) | | 1 | secure record |
| Same method of ascertainment for cases and controls(**Exposure**) | | 1 | yes |
| Non-Response rate(**Exposure**) | | 1 | same rate for both groups |

**Qiao et al. 2018**

| Study type | Case-control study | | |
| --- | --- | --- | --- |
| Participants | Patients undergoing OSAS  N:87 cases, 32 controls  Mean age in years (SD): 51.80 (8.10) case(moderate), 48.20 (9.90) case (severe), 50.10 (7.30) control.  Gender: 32 males/0 females case(moderate), 55 males/0 females case(severe), 32 males /0 females control.  Location: China | | |
| Comparison | Comparison: patients with sleep disorder vs. control  Case: OSAS patients  Control: normal control | | |
| Outcomes | Main study outcome: bone metabolic markers  Available outcomes: vitamin D levels of poor sleepers and good sleepers. | | |
| ***Risk of bias*** | | | |
| **Bias** | | **Authors’ judgment** | **Support for judgment** |
| Is the case definition adequate(**Selection**) | | 1 | yes, with independent validation |
| Representativeness of the cases(**Selection**) | | 1 | consecutive or obviously representative series of cases |
| Selection of Controls(**Selection**) | | 0 | hospital controls |
| Definition of Controls(**Selection**) | | 1 | no history of disease (endpoint) |
| Comparability of cases and controls on the basis of the design or analysis(**Comparability**) | | 2 | study controls for age, gender and other factors. |
| Ascertainment of exposure(**Exposure**) | | 1 | secure record |
| Same method of ascertainment for cases and controls(**Exposure**) | | 1 | yes |
| Non-Response rate(**Exposure**) | | 1 | same rate for both groups |
