# Supplementary material for: A meta‐analysis: Does vitamin D play a promising role in sleep disorders?
Source: Food Sci Nutr. 2020 Sep 9;8(10):5696–709. doi: 10.1002/fsn3.1867 (PMC7590291; doi:10.1002/fsn3.1867)
Supplement: Supplementary file 9 — TableS2 [file FSN3-8-5696-s009.docx]

**supplementation materials**

**Majid et al. 2017**

| Methods | RCT, (vitamin D vs. placebo)  8 weeks  Summary risk of bias: low | |
| --- | --- | --- |
| Participants | People aged 20–50 years with sleep disorders based on Pittsburg Sleep Quality Index (PSQI) referring to Golestan hospital Ahvaz.  N: 44 intervention, 45 control  Mean age in years (SD): 37.9(9.50) intervention, 35.50(10.00) control  Gender: 11 males/33females intervention, 10 males /35 females control  Location: Iran | |
| Interventions | Type: supplement (edible pearls)  Comparison: vitamin D supplementation vs. control  Intervention: Participants in intervention group received four edible pearls, each 50 000 IU vitamin D one in a fortnight.  Control: a placebo capsule was given one in a fortnight.  Compliance: At the entrance to study, general instructions were given to all participants not to change their diet by deleting dietary sources of vitamin D, changing physical activity pattern nor losing weight. At the beginning and the end of the study, a 3-day food record was taken (two consecutive days and a holiday).  Length of intervention: 8 weeks | |
| Outcomes | Main study outcome: food intake, serum vitamin D level, sun exposure, physical activity measure, daily nap sleep score and sleep subgroups score in the study groups at the beginning and the end of the study and intergroup comparison  Dropouts:2 intervention, 2 control  Available outcomes: PSQI score in the study group at the beginning and the end of the study and control group. | |
| Notes | The score of intervention group and control group, the beginning and the end of the intervention group were compared. | |
| ***Risk of bias*** | | |
| **Bias** | **Authors’ judgment** | **Support for judgment** |
| Random sequence generation (selection bias) | Low risk | This was a randomized clinical trial. |
| Allocation concealment (selection bias) | Low risk | This was a double-blinded clinical trial randomized by  a statistician. |
| Blinding of participants and personnel (performance bias)  All outcomes | Low risk | double-blinded |
| Blinding of outcome assessment (detection bias)  All outcomes | Low risk | Participants of the study, project  executives and clinic’s personnel were completely unaware of (blinded) control and intervention groups. |
| Incomplete outcome data (attrition bias)  All outcomes | Low risk | Participant flow well described. |
| Selective reporting (reporting bias) | Low risk | Clinical Trial Center by code no. 2015122725723N1IRCT |
| Attention | Low risk | All participants appear to have had similar frequency and quantity of attention and follow-up. |
| Compliance | Low risk | At the entrance to study, general instructions were given to all participants not to change their diet by deleting dietary sources of vitamin D, changing physical activity pattern nor losing weight. At the beginning and the end of the study, a 3-day food record was taken (two consecutive days and a holiday). |
| Other bias | Low risk | No commercial company involved, and no conflict of interest. |

**Ghaderi et al. 2017**

| Methods | RCT, (vitamin D vs. placebo)  12 weeks  Summary risk of bias: low | |
| --- | --- | --- |
| Participants | patients with maintenance methadone treatment(MMT) aged 25–70 years  N: 34 intervention, 34 control  Mean age in years (SD): 40.10(9.20) intervention, 42.50 (8.90) control  Location: Iran | |
| Interventions | Type: supplement (capsule)  Comparison: vitamin D supplementation vs. control  Intervention: supplemented with 50,000 IU of vitamin D every 2 weeks for 12 weeks  Control: supplemented with placebo every 2 weeks for 12 weeks  Compliance: To evaluate the compliance, serum 25(OH) vitamin D of patients was quantified. To ensure adherence, patients received a short message on their cell phones to intake the supplements daily. All patients completed 3-day food and three physical activity records as metabolic equivalents (METs) at weeks 0, 3, 6, 9 and 12 of the treatment.  Length of intervention: 12 weeks | |
| Outcomes | Main study outcome: Mental health parameters and metabolic status at baseline and after the 12-week intervention in maintenance methadone treatment subjects, adjusted changes in metabolic variables maintenance methadone treatment subjects that received either vitamin D supplements or placebo.  Dropouts: 0  Available outcomes: PSQI score in the intervention group at the beginning and the end of the study and control group. | |
| Notes | The score of intervention group and control group, the beginning and the end of the intervention group were compared. | |
| ***Risk of bias*** | | |
| **Bias** | **Authors’ judgment** | **Support for judgment** |
| Random sequence generation (selection bias) | Low risk | randomized double-blind placebo-controlled clinical trial |
| Allocation concealment (selection bias) | Low risk | Randomization assignment was done using computer-generated random numbers and was done by a trained staff at the clinic as blindness. |
| Blinding of participants and personnel (performance bias)  All outcomes | Low risk | double-blinded |
| Blinding of outcome assessment (detection bias)  All outcomes | Unclear risk | Not described. |
| Incomplete outcome data (attrition bias)  All outcomes | Low risk | Participant flow well described. |
| Selective reporting (reporting bias) | Low risk | registration of clinical trials:IRCT201701035623N102 |
| Attention | Low risk | All participants appear to have had similar frequency and quantity of attention and follow-up. |
| Compliance | Low risk | To evaluate the compliance, serum 25(OH) vitamin D of patients was quantified. To ensure adherence, patients received a short message on their cell phones to intake the supplements daily. All patients completed 3-day food and three physical activity records as metabolic equivalents (METs) at weeks 0, 3, 6, 9 and 12 of the treatment. |
| Other bias | Low risk | No commercial company involved, and no conflict of interest. |

**Wei et al. 2013**

| Methods | RCT, (the beginning and the end of the intervention group)  3 months  Summary risk of bias: high | |
| --- | --- | --- |
| Participants | All patients receive regular medical care and pain management.  N: 28intervention  Mean age in years (SD): 47.55(12.00) insufficient(INS) subgroup, 44.58( 9.46) deficient(DEF) subgroup.  Gender: 11 males/4females INS subgroup, 7 males /6 females DEF subgroup  Location: the U.S. | |
| Interventions | Comparison: the beginning and the end of the intervention group  Intervention: The INS subgroup was supplemented with vitamin D 1200 IU daily, and the DEF subgroup was supplemented with vitamin D 50,000 IU weekly.  Compliance: To evaluate the compliance, mostly over the phone (89.3%) but with a few face-to-face sessions.  Length of intervention: 3 months | |
| Outcomes | Main study outcome: Serum vitamin D response, pain response, sleep response and QoL response to vitamin D supplementation.  Dropouts: 0  Available outcomes: PSQI score at the beginning and the end of the intervention group. | |
| Notes | The PSQI score at the beginning and the end of the intervention group were compared. | |
| ***Risk of bias*** | | |
| **Bias** | **Authors’ judgment** | **Support for judgment** |
| Random sequence generation (selection bias) | Low risk | This study was designed prospectively. |
| Allocation concealment (selection bias) | Unclear risk | Not described |
| Blinding of participants and personnel (performance bias)  All outcomes | High risk | Obviously not used |
| Blinding of outcome assessment (detection bias)  All outcomes | High risk | Obviously not used |
| Incomplete outcome data (attrition bias)  All outcomes | Low risk | There were no dropout. |
| Selective reporting (reporting bias) | Unclear risk | The clinical registration number was lacked. |
| Attention | Low risk | No problem with attention bias. |
| Compliance | Low risk | To evaluate the compliance, mostly over the phone (89.3%) but with a few face-to-face sessions. |
| Other bias | Low risk | No commercial company involved, and no conflict of interest. |

**Mason et al. 2016**

| Methods | RCT, (vitamin D vs. placebo)  12 months  Summary risk of bias: low | |
| --- | --- | --- |
| Participants | postmenopausal women aged 50–75y who were overweight or obese.  N: 83 intervention, 84 control  Gender: 0 males/83females intervention, 0 males /84 females control  Location: the U.S. | |
| Interventions | Type: supplement (capsule)  Comparison: vitamin D supplementation vs. control  Intervention: oral vitamin D3 supplementation (cholecalciferol, 2000 IU/d)  Control: sunflower oil  Compliance: At randomization, participants received a 6-mo supply of study medications. Medication bottles were returned at 6 mo, and the remaining capsules were counted before a second 6-mo supply was provided. Likewise, at 12 mo, the second bottle and any  remaining capsules were returned and counted. Medication counts and the 12-mo change in serum vitamin D were used as indicators of study medication adherence.  Length of intervention: 12 months | |
| Outcomes | Main study outcome: 12-month changes in depressive symptoms, health-related quality of life (HRQOL), and sleep quality in women randomized to 2000IU/d vitamin D versus placebo.  Dropouts: 26 intervention, 25 control  Available outcomes: PSQI score in the intervention group at the beginning and the end of the study and control group. | |
| Notes | The score of intervention group and control group, the beginning and the end of the intervention group were compared. | |
| ***Risk of bias*** | | |
| **Bias** | **Authors’ judgment** | **Support for judgment** |
| Random sequence generation (selection bias) | Low risk | randomized clinical trial |
| Allocation concealment (selection bias) | Low risk | The random assignment was generated by a computerized program. |
| Blinding of participants and personnel (performance bias)  All outcomes | Low risk | double-blinded |
| Blinding of outcome assessment (detection bias)  All outcomes | Low risk | All other study staff was blind to the randomization status. |
| Incomplete outcome data (attrition bias)  All outcomes | Low risk | Participant flow well described. |
| Selective reporting (reporting bias) | Low risk | registration of clinical trials:NCT01240213 |
| Attention | Low risk | All participants appear to have had similar frequency and quantity of attention and follow-up. |
| Compliance | Low risk | At randomization, participants received a 6-mo supply of study medications. Medication bottles were returned at 6 mo, and the remaining capsules were counted before a second 6-mo supply was provided. Likewise, at 12 mo, the second bottle and any remaining capsules were returned and counted. Medication counts and the 12-mo change in serum vitamin D were used as indicators of study medication adherence. |
| Other bias | Low risk | No commercial company involved, and no conflict of interest. |
